# The Fibro-Inflammatory Ovary: Stromal Fibrosis, Extracellular Matrix Remodeling, and Mechanotransduction in Female Infertility

**DOI:** 10.3390/cimb48090917

**Published:** 2026-09-08

**Authors:** Charalampos Voros, Fotios Chatzinikolaou, Georgios Papadimas, Sofoklis Stavros, Ioannis Papapanagiotou, Nektaria Zagorianakou, Ali Can Gunes, Athanasios Karpouzos, Kyriakos Bananis, Charalampos Tsimpoukelis, Maria Anastasia Daskalaki, Stylianos Makrydimas, Nikolaos Thomakos, Panagiotis Antsaklis, Dimitrios Loutradis, Georgios Daskalakis

**Affiliations:** 1 Department of Obstetrics and Gynecology, ‘Alexandra’ General Hospital, National and Kapodistrian University of Athens, 80 Vasilissis Sofias Avenue, 11528 Athens, Greece; thanoskarpouzosdr@hotmail.com (A.K.); tsimpoukelischa@gmail.com (C.T.); md181341@students.euc.ac.cy (M.A.D.); thomakir@hotmail.com (N.T.); panosant@gmail.com (P.A.); gdaskalakis@yahoo.com (G.D.); 2 Laboratory of Forensic Medicine and Toxicology, School of Medicine, Aristotle University of Thessaloniki, 54124 Athens, Greece; 3 Athens Medical School, National and Kapodistrian University of Athens, 15772 Athens, Greece; dr.georgepapadimas@gmail.com (G.P.); gpapamd@hotmail.com (I.P.); loutradi@otenet.gr (D.L.); 4 Third Department of Obstetrics and Gynecology, Attikon Hospital, Medical School, National and Kapodistrian University of Athens, 10559 Athens, Greece; sfstavrou@yahoo.com; 5 Scientific Laboratory for Innovative Technologies in Internal Medicine, Preventive Medicine and Overall Care, Department of Nursing, School of Health Sciences, University of Ioannina, 45500 Ioannina, Greece; zagorianakou@uoi.gr; 6 Department of Obstetrics and Gynecology, Faculty of Medicine, Hacettepe University, Ankara 06100, Turkey; acgunes@hacettepe.edu.tr; 7 King’s College Hospitals NHS Foundation Trust, London SE5 9RS, UK; kyriakos.bananis@nhs.net; 8 Medical School, Aristotle University of Thessaloniki, 54124 Thessaloniki, Greece; stylianosmakrydimas@hotmail.com

**Keywords:** stromal fibrosis, infertility, extracellular matrix remodeling, in vitro fertilization, fibro-inflammatory ovary

## Abstract

Endocrine disorders and inherent flaws in oocyte quality are traditional causes of female infertility. Increasing research indicates that structural and biomechanical alterations in the ovarian microenvironment may significantly contribute to reproductive decline. Progressive extracellular matrix remodeling, stromal fibrosis, chronic inflammation, and heightened tissue stiffness seem to affect follicular homeostasis, granulosa–oocyte communication, and ovarian function. Fibro-inflammatory pathways, including transforming growth factor-β (TGF-β), Hippo/YAP-TAZ signaling, oxidative stress, macrophage activation, and mechanotransduction, are increasingly associated with ovarian ageing, polycystic ovary syndrome, reduced ovarian reserve, and unfavourable reproductive outcomes. Here, the term ‘fibro-inflammatory ovary’ is used descriptively to unify these mechanisms rather than as an established clinical entity. Mechanical alterations of the ovarian stroma may affect follicular activation, vascularization, and oocyte competence by altering cellular tension and extracellular matrix dynamics. Our review examines growing findings about ovarian fibrosis and stromal remodeling in female infertility, focusing on molecular causes, mechanobiology, and translational implications for assisted reproduction. Potential diagnostic uses, such as ovarian elastography, and prospective anti-fibrotic therapy techniques are also examined.

## 1. Introduction

Female infertility is a major global health issue, impacting millions of women of reproductive age globally and significantly increasing the need for assisted reproductive technology. Conventional contemporary theories of reproductive failure have emphasized endocrine dysfunction, compromised folliculogenesis, chromosomal anomalies, and inherent deficiencies in oocyte competence [1]. Reduced ovarian reserve, mitochondrial dysfunction, oxidative stress, and age-associated meiotic instability are increasingly acknowledged as contributors to reproductive ageing and suboptimal IVF results. Notwithstanding substantial progress in reproductive endocrinology and embryology, up to one-third of couples attending tertiary infertility clinics remain without an identifiable cause on standard hormonal or follicular criteria alone, representing a substantial unmet clinical need that the fibro-inflammatory framework proposed in this review is intended to address [2].

We argue that a substantial share of this unexplained reproductive failure reflects a common, underrecognized process, the progressive conversion of the ovarian stroma into a stiff, fibro-inflammatory tissue, that operates across strikingly different clinical conditions and constitutes a unifying mechanism rather than a disease-specific curiosity.

The ovarian microenvironment and its function in sustaining follicular homeostasis have garnered heightened interest lately. Ovarian physiology relies on coordinated endocrine signaling as well as the structural and biochemical integrity of the surrounding stromal compartment [3]. The developing oocyte, granulosa cells, vascular networks, immune cells, fibroblasts, and extracellular matrix (ECM) components interact dynamically to establish a highly specialized niche that facilitates follicular development and maturation. Alterations in this microenvironment may significantly affect ovarian function far in advance of the clinical manifestation and quantification of follicular pool depletion [4]. Over the past decade, advances in single-cell transcriptomics, proteomics, and tissue elastography have repositioned ovarian fibrosis from an incidental histological observation to a candidate driver of reproductive decline, prompting closer scrutiny of its underlying mechanisms.

The aging-related remodeling of the ovarian stroma has been a significant focus for research. Ageing ovaries exhibit a gradual buildup of collagen fibres, cross-linking of the extracellular matrix, activation of fibroblasts, and continuous low-grade inflammation. Comparable fibro-inflammatory alterations have also been documented in polycystic ovary syndrome (PCOS), infertility linked to obesity, endometriosis, reduced ovarian reserve, and premature ovarian insufficiency [5]. Ovarian fibrosis is increasingly recognized as a dynamic biological process that actively modifies follicular architecture, tissue vascularization, immunological signaling, and cellular communication, rather than representing a static end-stage of scar accumulation [6].

Chronic inflammation exacerbates stromal remodeling and fibrosis in the ovary. Activated macrophages, inflammasome signaling, oxidative stress, TGF-β, and pro-fibrotic cytokines provide a self-sustaining fibro-inflammatory milieu that may disrupt granulosa-oocyte communication and follicular development [7]. Recent research indicates that ovarian stiffness may be quantifiable by elastographic imaging techniques. There exists the possibility to create innovative biomarkers that may indicate biological ovarian ageing irrespective of chronological age [8].

Despite this accumulating evidence, several fundamental questions remain unresolved. It is not yet established whether ovarian fibrosis is a cause of follicular depletion and reproductive decline, a downstream consequence of it, or a parallel process driven by shared upstream triggers. The molecular drivers that initiate and sustain fibrotic progression across different reproductive pathologies, PCOS, endometriosis, obesity, and premature ovarian insufficiency have largely been characterized in isolation, without a clear account of whether they converge on a common mechanism or represent genuinely distinct pathways. Whether established ovarian fibrosis is reversible in humans, as opposed to in animal models, remains unknown, as does the extent to which emerging anti-fibrotic and mechanotransduction-targeted interventions can be translated into clinically meaningful reproductive benefit. This review addresses these four questions directly, evaluating the current evidence for causality, molecular convergence, reversibility, and translational potential in turn.

**Figure 1:**
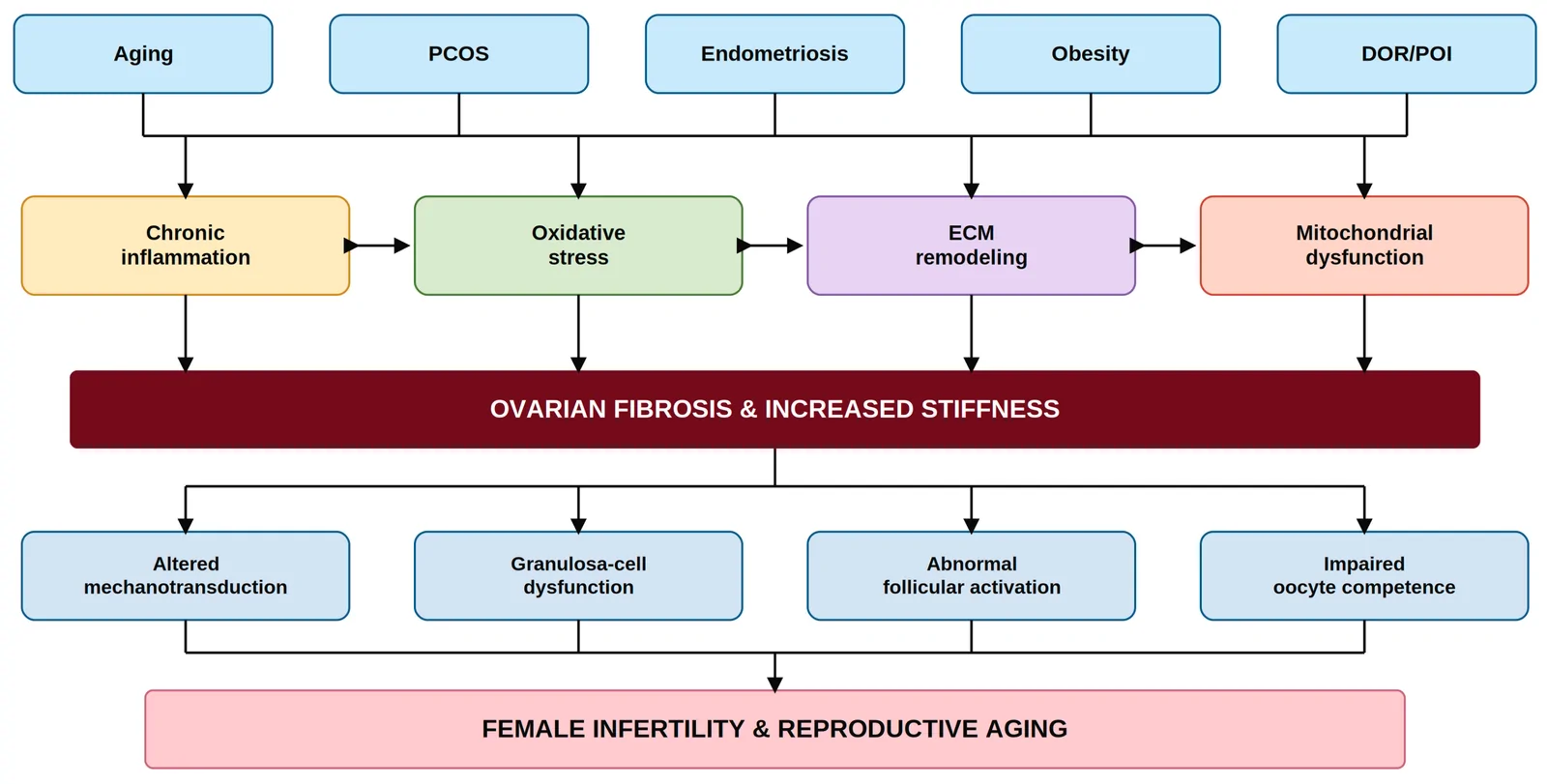
Integrated Fibro-Inflammatory Model of Ovarian Aging and Female Infertility. Schematic depiction of the interrelated pathways contributing to ovarian fibrosis and reproductive impairment. Chronic inflammation, oxidative stress, mitochondrial dysfunction, metabolic dysregulation, and extracellular matrix modification together contribute to stromal fibrosis and heightened ovarian stiffness. Altered ovarian biomechanics subsequently influence mechanotransduction pathways, granulosa cell functionality, follicular activation, and oocyte competence, finally leading to reproductive ageing and infertility. PCOS, endometriosis, obesity-related infertility, reduced ovarian reserve, and premature ovarian insufficiency exhibit common fibro-inflammatory and biomechanical mechanisms in the ovarian microenvironment. Abbreviations: PCOS, polycystic ovary syndrome; DOR, diminished ovarian reserve; POI, premature ovarian insufficiency; ECM, extracellular matrix.

Ovarian fibrosis, extracellular matrix remodeling, and mechanobiology in female infertility have recently been the subject of significant research, which is summarized in this review. We focus on molecular mechanisms that influence fibro-inflammatory remodeling, alterations in ovarian biomechanics, and the translational significance of stromal dysfunction in relation to reproductive ageing and assisted reproduction [9]. The evaluation also encompasses potential diagnostic uses and future anti-fibrotic treatment techniques, summarized schematically in the Fibro-Inflammatory Model of Ovarian Aging and Female Infertility presented in Figure 1.

## 2. Materials and Methods

We conducted an extensive narrative review of the literature utilizing the PubMed/MEDLINE electronic database to identify experimental, translational, and clinical studies concerning ovarian fibrosis, extracellular matrix remodeling, stromal inflammation, ovarian biomechanics, and mechanotransduction pathways in female infertility. We conducted a comprehensive analysis of the literature from January 2000 to May 2026, including earlier seminal research when considered crucial for understanding ovarian stromal biology and reproductive ageing.

Search criteria were selected to include traditional fibrosis-related pathways and novel notions in ovarian mechanobiology. Boolean operators (“AND”, “OR”) were employed in various combinations, tailored to the thematic emphasis of each search, to integrate the terms “ovarian fibrosis,” “ovarian stromal fibrosis,” “fibro-inflammatory ovary,” “ovarian stiffness,” “extracellular matrix ovary,” “mechanobiology ovary,” “Hippo signaling ovary,” “YAP/TAZ ovary,” “stromal remodeling infertility,” “ovarian inflammation,” “ovarian ageing fibrosis,” “female infertility,” “oocyte competence,” and “IVF.” Reference lists of chosen studies and current reviews were manually examined to discover other relevant articles that were not included in the first search procedure.

Eligible for inclusion were original research publications, translational studies, human observational studies, experimental animal models, and review articles directly pertaining to ovarian fibrosis and stromal remodeling. Priority for detailed discussion and data extraction was given to original research, translational, and experimental studies, whereas review articles were used primarily to provide contextual and thematic framing rather than as a substitute for primary evidence. Particular focus was placed on research concerning extracellular matrix deposition, inflammatory remodeling, ovarian stiffness, mechanotransduction signaling, Hippo/YAP-TAZ activation, oxidative stress, macrophage-mediated inflammation, and age-related stromal alterations. Experimental studies on ovarian ageing, polycystic ovary syndrome, obesity-related infertility, diminished ovarian reserve, premature ovarian insufficiency, and endometriosis-associated ovarian dysfunction were deemed particularly pertinent due to their strong association with fibro-inflammatory remodeling of the ovarian microenvironment. Articles concerning fibrosis in non-reproductive tissues, which were not directly relevant to the ovary, were omitted from the final synthesis. We eliminated conference abstracts without accessible full-text articles, duplicate publications, non-English research, and those without mechanistic or reproductive relevance. For studies involving human ovarian tissue specifically, we additionally excluded case reports and case series with fewer than three subjects, studies lacking histological or immunohistochemical confirmation of the reported fibrotic or stromal changes, and studies in which the diagnostic criteria used to define the clinical population, such as polycystic ovary syndrome, diminished ovarian reserve, premature ovarian insufficiency, or endometriosis, were not stated.

Initially, titles and abstracts were evaluated for relevance, followed by a comprehensive assessment of the chosen papers in their entirety. We focused on molecular pathways pertinent to stromal fibrosis, extracellular matrix remodeling, inflammatory signaling, tissue biomechanics, and mechanotransduction for data extraction. We also emphasized research on the correlation between ovarian fibrosis and follicular development, granulosa–oocyte communication, ovarian reserve, oocyte competence, and assisted reproductive outcomes. The evaluation included elastographic assessment of ovarian stiffness and experimental anti-fibrotic treatment techniques as novel diagnostic methods.

There was significant variability across the research on experimental design, molecular methodologies, tissue characterization techniques, and reproductive outcomes. Consequently, a quantitative synthesis was deemed unsuitable. Findings were synthesized using a narrative mechanistic method to critically examine the interaction among fibrosis, inflammation, extracellular matrix dynamics, ovarian biomechanics, and reproductive failure. Significant emphasis was focused on recent research indicating that ovarian fibrosis is an active and physiologically dynamic process that may alter follicular architecture and ovarian function, rather than being a purely passive result of ageing tissue.

## 3. The Healthy Ovarian Stroma and Extracellular Matrix

### 3.1. The Ovarian Stroma as a Functional Biological Compartment

Ovarian physiology has traditionally been seen mostly from a follicle-centric perspective. Traditional reproductive models have mostly concentrated on endocrine control, granulosa cell function, gonadotropin responsiveness, and inherent factors influencing oocyte competence. However, a more intricate understanding of ovarian biology has emerged throughout time. Increasing data indicates that follicular formation cannot be comprehended apart from the surrounding stromal environment [10]. The anatomical arrangement of ovarian tissue seems to be intricately linked to follicular activity, cellular metabolism, vascular support, inflammatory equilibrium, and the maintenance of reproductive function. The ovarian stroma is not only a passive connective structure. It is a physiologically active compartment that experiences ongoing remodeling and substantial cellular communication [11].

The ovarian microenvironment is defined by interactions among many cell types. Stromal fibroblasts, endothelial cells, immunological populations, pericytes, granulosa cells, theca cells, and extracellular matrix components collaborate to maintain tissue homeostasis throughout reproductive life [12]. Communication across these compartments transpires via direct cell–cell contacts, soluble cytokines, growth factors, extracellular vesicles, and mechanically transmitted signals derived from the tissue architecture. The expanding understanding of this intricate stromal ecology has significantly altered the prevailing views on reproductive ageing and ovarian failure.

The extent of specialization in stromal control is also evident in the spatial arrangement of ovarian tissue. Primordial follicles are mostly situated in the ovarian cortex, marked by extensive collagen deposition, limited vascularity, and heightened tissue stiffness, with histological quantification of human ovarian cortical biopsies confirming collagen occupying over half of the tissue area (55.5 ± 1.7%) alongside high stromal cell density (3.6 ± 0.2 × 10^6^ cells/mm^3^) [13]. Preserving this mechanically constrained environment is essential for sustaining follicular dormancy and averting premature activation of the primordial follicle reservoir. The advancement of follicular maturation correlates with a migration to deeper medullary regions, where the extracellular matrix composition, vascular supply, oxygen availability, and tissue elasticity significantly vary from those of the ovarian cortex [14].

Ovarian fibroblasts not only provide structural support but also actively engage in cytokine production, wound healing, angiogenesis control, and inflammatory signaling [15]. In one such experimental characterization, fibroblasts isolated from 14 normal ovarian tissue samples and 29 cell lines derived from 5 ovarian tumor samples displayed distinct morphological and biochemical profiles under three-dimensional culture conditions, sorting reproducibly into normal, primed, and activated stromagenic stages that reflected their tissue of origin [15]. Follicular growth imposes considerable mechanical and metabolic stress on the surrounding stromal compartment, which continuously remodels its extracellular matrix to support the rapid development of the follicles. The adaptive process may, however, fail, leading to excessive collagen buildup, fibroblast activation, and tissue rigidity. In many pathological reproductive disorders, these alterations become more noticeable as reproductive age increases [16].

Furthermore, stromal integrity seems to be intricately associated with vascular homeostasis. Coordinated angiogenesis is essential for growing follicles to ensure oxygen supply, steroidogenesis, and nutrient transfer. Extracellular matrix proteins modulate endothelial cell migration and vessel stability, whereas stromal fibroblasts and macrophages participate in local angiogenic signaling, with endothelial cell proliferation accounting for 25–30% of proliferating cells in the inner thecal layer during the late secondary and tertiary stages of follicular development [17]. Deterioration of the stromal architecture over time may impair reproductive function via vascular and mechanical pathways. The acknowledgement of the ovarian stroma as a dynamic regulatory system, rather than a mere passive supporting tissue, has resulted in a paradigm change in reproductive biology. Structural alterations in the ovarian microenvironment may precede detectable endocrine disorders and may directly contribute to diminished fertility, decreased ovarian response, and compromised oocyte quality [13].

### 3.2. Extracellular Matrix Dynamics in the Ovary

Ovarian extracellular matrix architecture undergoes dynamic remodeling during reproductive life span. Cyclic follicular development, ovulation, luteal creation, and tissue repair need continuous modification of the extracellular environment to maintain structural stability while ensuring functional flexibility [13]. The ovarian extracellular matrix functions as a dynamic signaling platform that may affect cellular differentiation, migration, proliferation, and metabolic activity. Follicle development and ovarian tissue mechanics are both impacted by the biochemical make-up and local distribution of extracellular matrix proteins [4].

The primary structural constituents of the ovarian extracellular matrix are collagens. Types I and III collagen are abundantly present in the stromal compartment and play a crucial role in tissue stiffness and tensile strength, whereas type IV collagen is mostly found in follicular basement membranes [18]. Laminins, fibronectin, elastin, proteoglycans, and glycosaminoglycans contribute to the establishment of a highly organized three-dimensional network around follicles and vascular systems. Their distribution varies dynamically with follicular stage, hormonal environment, and reproductive age, indicating a strong correlation between the composition of the extracellular matrix and ovarian physiology [19]. This hierarchy is stage-dependent: collagen IV and laminin dominate the follicular basement membrane from the earliest primordial stages, whereas fibronectin becomes prominent only later, appearing consistently in the basement membrane of primary and secondary follicles but largely absent from primordial follicles. With age, this composition shifts further, with collagen and laminin content increasing progressively from prepuberty to menopause while elastin rises moderately through the reproductive years before declining sharply after menopause, changes that together track the transition from a permissive to an increasingly rigid follicular microenvironment [19].

Basement membranes encasing granulosa cells play crucial regulatory functions in follicular growth. The structural integrity of these membranes maintains the follicular architecture while concurrently controlling selective molecular exchange between the follicular compartment and the adjacent stromal tissue [20]. Laminin-rich basement membranes influence granulosa cell polarity, survival, proliferation, and steroidogenic activity via integrin-mediated signaling pathways. Disruption of basement membrane organization may directly affect granulosa cell function and hinder the support mechanisms for oocytes essential for meiotic competence [21].

A significant element of the ovarian extracellular matrix is the glycosaminoglycan hyaluronan, which plays crucial roles in reproduction. Elevated concentrations of hyaluronan accumulate in the cumulus–oocyte complex during the periovulatory phase, facilitating matrix hydration, cumulus expansion, and cellular communication around the oocyte. Hyaluronan modulation seems to be crucial for regular ovulatory functions and fertilisation capability. Alterations in hyaluronan metabolism have been associated with inflammatory remodeling, fibrosis, and impaired follicular dynamics, especially with ageing ovaries and metabolic reproductive diseases [22].

A meticulously maintained equilibrium between matrix formation and proteolytic breakdown is essential for the preservation of extracellular matrix homeostasis. A significant portion of this remodeling process is regulated by matrix metalloproteinases (MMPs) and their tissue inhibitors (TIMPs) [23]. Among the ovarian-relevant isoforms, MMP-2 and MMP-9 (gelatinases) are principally responsible for degrading collagen IV and other basement membrane components during follicular rupture and tissue remodeling, while TIMP-1 and TIMP-2 constrain this activity by directly inhibiting MMP catalytic function; disruption of this balance toward relatively excessive TIMP inhibition or insufficient MMP activity favors net collagen accumulation and fibrotic stiffening rather than physiological turnover. This imbalance has direct human evidence: women with PCOS show significantly elevated MMP-9 alongside reduced MMP-2, MMP-14, and TIMP-2 compared with healthy controls, a proteolytic–antiproteolytic shift consistent with impaired matrix turnover and progressive stromal fibrosis.

The disintegration of the extracellular matrix is meticulously managed, facilitating follicular enlargement, vascular invasion, tissue remodeling, and finally, ovulation. In order to facilitate oocyte release, proteolytic pathways are momentarily activated during the periovulatory phase, which weakens the follicular wall locally. Healthy ovarian tissue has extraordinary healing capacity, as seen by the quick restoration of extracellular matrix integrity following ovulation [24].

If this intricate remodeling mechanism is disrupted, the ovarian microenvironment may gradually become mechanically stiff and fibrotic. Excessive collagen accumulation, inadequate matrix breakdown, chronic inflammatory activation, and fibroblast dysregulation gradually modify the architecture of the extracellular matrix and compromise normal tissue elasticity. The persistent accumulation of crosslinked collagen fibres may hinder oxygen transport and vascular organization, leading to regions of metabolic stress in the ovarian cortex. Comparable anomalies of the extracellular matrix have been shown in ovarian ageing, polycystic ovary syndrome, obesity-related infertility, and endometriosis-related ovarian dysfunction [25].

The mechanical characteristics of extracellular matrix proteins influence ovarian cell behaviour via varied cellular signaling. Integrins provide a physical connection between extracellular matrix components and intracellular cytoskeletal networks, allowing mechanical inputs to influence transcriptional activity, mitochondrial metabolism, stress responses, and cellular differentiation. Among the ovarian integrin repertoire, α6β1 mediates laminin-driven granulosa cell survival, proliferation, and cytoskeletal reorganization, while β1 integrin shows broad expression across oocytes, granulosa cells, and theca cells throughout follicular development, positioning these receptors as the principal mechanical transducers linking basement membrane composition to granulosa cell behaviour [26,27,28,29,30,31,32].

### 3.3. Immune Homeostasis and Physiological Inflammation in the Ovary

Normal ovarian physiology is defined by the continual interplay between endocrine signals and meticulously managed inflammatory processes. Cyclic tissue remodeling linked to follicular development, ovulation, luteal transformation, and follicular atresia induces recurrent periods of cellular stress, extracellular matrix degradation, and temporary immune activation throughout reproductive life [33]. Unlike chronic pathological inflammation, physiological inflammation in the ovary is spatially confined and temporally regulated, resulting in tissue restructuring without enduring harm to the tissue structure.

Resident immune populations are essential for sustaining ovarian homeostasis. Macrophages constitute a predominant population of leukocytes inside the ovarian stroma, performing roles in angiogenesis, extracellular matrix turnover, phagocytosis of apoptotic cells, steroidogenesis, and tissue repair [34]. The functional diversity of ovarian macrophages seems to be particularly essential for sustaining the inflammatory equilibrium. Reparative macrophage phenotypes participate in reorganization and vascular stability, while excessive activation of pro-inflammatory pathways may lead to oxidative stress, fibroblast activation, and collagen deposition [35].

Additionally, neutrophils, dendritic cells, mast cells, and T lymphocytes participate in the immunological control of the ovaries. The dynamics of immune-cell populations during the menstrual cycle are unpredictable and seem to be intimately associated with hormonal variations and the time of ovulation [36]. Among these, mast cells occupy a distinct mechanistic role: tryptase released upon their activation drives collagen I production in ovarian theca–stromal cells through protease-activated receptor-2 signaling, a pathway directly implicated in the fibrotic remodeling seen in premature ovarian insufficiency. Ovulation resembles a localised inflammatory response marked by heightened vascular permeability, leukocyte infiltration, prostaglandin synthesis, cytokine secretion, and proteolytic activity. Follicular rupture and oocyte release need a temporary degradation of the extracellular matrix at the follicular apex, followed by rapid reparative processes that reinstate tissue integrity [37]. With advancing reproductive age, this resolution process becomes progressively less efficient. Repeated cycles of ovulatory injury and repair are thought to leave a cumulative inflammatory signature, contributing to the transition toward a chronically pro-inflammatory ovarian microenvironment, or ‘inflammaging,’ characterized by altered immune cell composition and sustained elevation of inflammatory mediators rather than their timely resolution after each cycle.

Physiological ovulation includes inflammatory mediators, including interleukins, tumour necrosis factor-α, prostaglandins, chemokines, reactive oxygen species, and TGF-β. Under typical conditions, these pathways are rigorously controlled and self-restraining [38]. However, the prolonged persistence of analogous inflammatory signals may gradually transform the ovarian milieu into a fibro-inflammatory state. The continual stimulation of macrophages and stromal fibroblasts may lead to enhanced extracellular matrix deposition and increasing tissue stiffness.

A further link between inflammation and stromal remodeling is oxidative stress. Chronic inflammatory activation throughout reproductive life exposes ovarian tissue to cumulative oxidative damage, potentially harming mitochondria, proteins, lipids, and extracellular matrix components. An overabundance of reactive oxygen species (ROS) may activate pro-fibrotic signaling pathways and impair granulosa cell contact with the oocyte. Therefore, it is possible that reproductive ageing is accelerated by the slow buildup of oxidative and inflammatory damage [39].

Tissue biomechanics, immunological signaling, and the extracellular matrix seem to be more and more interdependent on one another. Inflammatory cytokines regulate fibroblast activity and matrix remodeling. The rigidity of the extracellular matrix may amplify inflammatory activation via mechanosensitive signaling pathways. Fibrosis, inflammation, oxidative stress, and biomechanical dysfunction may all feed into each other in a vicious cycle that can be set up by increasing stromal remodeling [40].

The increasing acknowledgement of the ovary as an immunologically active organ has significantly expanded contemporary perspectives on female reproductive biology. Alongside sufficient hormonal support, the preservation of reproductive competence requires the maintenance of inflammatory equilibrium, structural integrity, vascular homeostasis, and extracellular matrix adaptability. Disturbance of this fragile equilibrium may signify a first biological occurrence that contributes to ovarian senescence and infertility prior to the clinical manifestation of complete follicular depletion.

## 4. Ovarian Fibrosis and Reproductive Aging

### 4.1. Age-Associated Stromal Remodeling in the Ovary

Reproductive ageing has traditionally been seen primarily as a gradual depletion of the primordial follicle reserve, accompanied by a decline in oocyte competence. Contemporary information increasingly indicates that ovarian ageing cannot be solely attributed to follicular depletion. Ovarian reserve depletion may not be clinically apparent for quite some time, but structural degradation of the ovarian microenvironment appears to happen gradually throughout reproductive life and may actively contribute to diminishing fertility [41]. Ageing in murine models is marked by significant remodeling of the stromal tissue, including collagen buildup, disorganization of the extracellular matrix, fibroblast activation, chronic inflammatory infiltration, and tissue stiffness [13]. Parallel, though independently derived, evidence in human ovarian cortical tissue similarly shows a progressive increase in collagen and stiffness from the reproductive years onward [13], suggesting that comparable stromal ageing processes occur across species, although this has not been demonstrated within a single study directly comparing murine and human tissue.

During reproductive senescence, significant structural alterations gradually transpire in both the medullary and cortical areas of the ovary. In the ovarian cortex, dense aggregates of collagen develop, and the turnover of the extracellular matrix becomes more dysregulated due to an imbalance between matrix production and breakdown. Concurrently, the natural flexibility of the stroma progressively diminishes due to cross-linking and the excessive accumulation of collagen fibres [22]. Fibrotic remodeling also modifies tissue hydration, extracellular matrix alignment, and biomechanical architecture of the ovarian niche. As stromal flexibility declines, it may provide a mechanically constrained environment that hinders follicular growth, vascular adaptability, and coordinated tissue remodeling during folliculogenesis. Alterations in the structure of this magnitude significantly alter the physical environment in which follicles mature [3].

Vascular remodeling is a significant characteristic of stromal ageing. Ageing ovaries are often marked by reduced microvascular density, compromised angiogenic responses, endothelial dysfunction, and diminished perfusion of the cortical and medullary regions. Optimal vascular support for follicular growth is essential for ensuring oxygen supply, steroidogenesis, nutrient transport, and metabolic balance [42]. Disruption of vascular architecture may create localized hypoxic conditions that might exacerbate oxidative stress, inflammatory signaling, and fibroblast activation. Progressive deterioration of tissue perfusion may further jeopardize the fragile energy equilibrium necessary for oocyte maturation and hinder granulosa cell metabolism [43].

The behaviour of fibroblasts is significantly modified throughout reproductive ageing and seems to be essential for the progression of ovarian fibrosis. Senescent fibroblasts have a distinctive secretory phenotype characterized by elevated production of inflammatory cytokines, TGF-β, extracellular matrix proteins, matrix-remodeling enzymes, and pro-fibrotic mediators [7]. The persistent release of these chemicals gradually alters the ovarian milieu into a persistently remodeling fibro-inflammatory niche. Furthermore, activated fibroblasts have modified reactions to mechanical and inflammatory stimuli, resulting in persistent collagen accumulation and heightened tissue stiffness. Ovarian ageing may therefore be caused by the persistent buildup of these defective stromal cells rather than being only an effect of reproductive ageing [6].

Cellular senescence extends beyond stromal fibroblasts, impacting many ovarian compartments concurrently. Granulosa cells, endothelial cells, immunological populations, and stromal support cells exhibit age-associated mitochondrial dysfunction, oxidative damage, impaired proteostasis, telomere attrition, and modified metabolic activity [44]. Senescent stromal cells may affect adjacent follicles and impair reproductive success by releasing inflammatory mediators, reactive oxygen species, and extracellular vesicles containing stress-related molecular components. Follicular degeneration occurs within a progressively adverse milieu marked by persistent cellular stress and structural instability [45].

Recently, the mechanical ramifications of stromal ageing have garnered heightened scientific attention. The gradual hardening of the extracellular matrix modifies local tissue tension and disrupts mechanosensitive signaling pathways essential for proper follicular activation and development. Overly stiff cortical settings may trigger aberrant activation dynamics, hinder granulosa-cell proliferation, and disrupt metabolic connection with the oocyte in primordial follicles, consistent with direct measurements showing that ovarian stiffness rises progressively with age and tracks increased collagen and reduced hyaluronan content in the stroma [22]. Excessive collagen deposition may lead to mechanical constraints that hinder the capacity of growing follicles to adjust to varying biomechanical requirements by obstructing follicular growth during later maturation phases. Results like these lend credence to the idea that biomechanics play a crucial role in ovarian ageing, in addition to biochemistry and steroids [26].

Experimental investigations have shown a robust correlation between diminished reproductive function and stromal fibrosis. Progressive increases in ovarian stiffness have been consistently seen in mouse models of reproductive ageing, accompanied by heightened collagen deposition, inflammatory activation, macrophage infiltration, and diminished fertility [22]. Significantly, many experimental interventions targeting fibrosis-related pathways have shown partial repair of ovarian structure and enhancement of reproductive metrics. Preclinical investigations have shown that anti-fibrotic therapies may diminish collagen accumulation or alter extracellular matrix remodeling, positively impacting follicular development and ovarian function [25]. Instead of being an inevitable consequence of ovarian decline, our findings indicate stromal fibrosis is a dynamic biologic component of reproductive ageing that may be amenable to modification.

### 4.2. Inflammaging and Chronic Fibro-Inflammatory Signaling

Chronic inflammatory activation seems to have a significant role in the progression of ovarian fibrosis. Chronic low-grade inflammation (inflammaging) is acknowledged as a primary biological characteristic of reproductive senescence. The ovary experiences repeated inflammatory remodeling throughout reproductive life, influenced by ovulation, follicular rupture, extracellular matrix degradation, luteal regression, and tissue healing [5]. Physiological inflammatory reactions during early reproductive years are well regulated and self-limiting. Ageing gradually alters this equilibrium, leading to sustained activation of inflammatory pathways that may induce stromal damage and fibrotic remodeling. The accumulation of inflammatory damage over decades may profoundly alter the ovarian microenvironment [46].

Macrophage infiltration of the ageing ovarian stroma is one of the first and most constant characteristics of ovarian inflammaging. Age-associated alterations in macrophage phenotype result in heightened synthesis of tumour necrosis factor-α, interleukin-1β, interleukin-6, TGF-β, and other chemotactic mediators that may provoke fibroblast activation and extracellular matrix accumulation [47]. Thus, sustained macrophage-mediated inflammation may directly facilitate increasing collagen deposition and stromal rigidity. Furthermore, persistent innate immune signaling induces localized oxidative stress and tissue remodeling, establishing a self-sustaining inflammatory milieu. Comparable inflammatory markers have also been documented in naturally aged ovaries and in many infertility-related diseases [48].

The activation of nuclear factor-kappa B signalling plays a pivotal role in ovarian inflammaging. In the ovarian stroma, this pathway is triggered by advanced glycation end-products acting through their receptor RAGE, by damage-associated molecular patterns released from senescent or stressed cells acting through NLRP3, and by reactive oxygen species generated during chronic oxidative stress. Persistent activation of NF-κB through these stimuli leads to the production of inflammatory cytokines, matrix-remodeling enzymes, adhesion molecules, and pro-fibrotic mediators, which may exacerbate existing stromal damage. Oxidative stress and mitochondrial dysfunction are exacerbated by the continuous production of reactive oxygen species and damage-associated molecular patterns released by stressed or senescent cells [49]. Chronic NF-κB activation influences macrophage polarisation, fibroblast differentiation, and extracellular matrix turnover, therefore directly associating inflammatory activity with the progression of fibrosis. The persistent activation of these pathways progressively transforms the ovarian stroma into a fibro-inflammatory milieu with diminished regeneration capacity [50].

TGF-β is a primary molecular mediator linking inflammation and fibrosis. The activation of TGF-β signalling is a continuous process that enhances fibroblast proliferation, collagen production, and extracellular matrix cross-linking, and inhibits matrix breakdown processes. TGF-β concurrently affects immune cell differentiation and fosters the development of a persistently inflammatory tissue milieu [51]. The heightened activity of TGF-β has been linked to several systemic fibrotic disorders and is gaining significance in relation to ovarian ageing and infertility. Consequently, persistent stimulation of this pathway may serve as a principal catalyst for age-related stromal remodelling [52].

Inflammaging significantly impacts ovarian vascular integrity and metabolic equilibrium. Chronic inflammatory activation causes endothelial cell dysfunction, diminishes angiogenic efficacy, and results in microvascular remodeling. Decreased perfusion may induce areas of persistent hypoxia in the ovarian cortex, thereby exacerbating oxidative stress and pro-fibrotic signaling. Hypoxic microenvironments influence granulosa cell metabolism and may hinder oxygen-dependent activities essential for optimal oocyte maturation and meiotic development. Inflammation, vascular dysfunction, oxidative damage, and fibrosis may interact with one another to contribute to ovarian decline [53].

Recurrent ovulatory cycles may inherently lead to chronic inflammatory load in the ovary. Ovulation resembles a localized inflammatory response characterized by the synthesis of prostaglandins, recruitment of leukocytes, proteolytic activity, destruction of the extracellular matrix, and generation of reactive oxygen species [37]. Physiological healing processes are often enough to efficiently restore tissue integrity. Prolonged exposure to recurrent inflammatory remodeling over decades may progressively promote chronic fibro-inflammatory activation. Even in the absence of obvious clinical illness, reproductive ageing may be accelerated, and these pathways might provide some insight into why [54].

Numerous infertility-related illnesses have common inflammatory and fibrotic characteristics. Obesity, metabolic syndrome, endometriosis, polycystic ovary syndrome, and reduced ovarian reserve exhibit varying levels of chronic inflammatory dysfunction, characterised by extracellular matrix abnormalities and stromal remodelling. The fact that inflammatory and pro-fibrotic pathways are involved in all of these diseases points to chronic fibro-inflammatory signaling as a shared biological mechanism that may contribute to a number of reproductive dysfunctions.

### 4.3. Oxidative Stress and Fibrosis Amplification

Oxidative stress is a primary molecular mechanism connecting reproductive ageing with the development of stromal fibrosis. Physiological concentrations of reactive oxygen species have been associated with ovulation, steroidogenesis, intracellular signaling, and follicular maturation. Under typical circumstances, the antioxidant mechanisms in the ovaries preserve redox equilibrium and avert excessive oxidative harm [6]. This distinction is, however, difficult to draw precisely in practice: ovarian reactive oxygen species production spans a continuum from tightly controlled redox signaling that supports physiological processes to unchecked excess that damages biomolecules, and no fixed concentration threshold reliably separates the two states across the different cellular compartments and follicular stages involved [54]. This ambiguity complicates efforts to define at what point age-related increases in oxidative burden shift from a normal feature of ovarian cycling to a pathological driver of fibrosis. Ageing increasingly disturbs homeostasis owing to the buildup of mitochondrial dysfunction, compromised antioxidant defence, chronic inflammatory activation, and metabolic instability. Subsequently, persistent oxidative stress induces inflammatory and pro-fibrotic signaling within the ovarian milieu, leading to the structural degradation of the ovarian stroma [55].

The primary locations of reactive oxygen species generation in ovarian tissue are the mitochondria inside the cells. The decline in oxidative phosphorylation associated with ageing gradually reduces the efficiency of the electron transport chain and increases electron leakage, resulting in the overproduction of reactive oxygen species, with human ovarian tissue showing a significant age-related increase in ROS-induced lipid peroxidation markers within oocytes and granulosa cells [56]. Oocytes are particularly susceptible to mitochondrial malfunction due to their significant energy requirements and dependence on precisely regulated ATP synthesis during meiotic maturation and early embryonic development. Mitochondrial anomalies in granulosa cells may further undermine metabolic support to the oocyte and intercellular communication within the follicular environment [57].

Reactive oxygen species actively influence extracellular matrix remodeling via many linked routes. Oxidative stress induces fibroblast activation, collagen production, extracellular matrix cross-linking, and TGF-β signaling, while simultaneously disrupting matrix breakdown pathways [58]. Oxidative alteration of extracellular matrix proteins may modify collagen architecture and facilitate the creation of stiff collagen networks that defy normal proteolytic turnover. Progressive stiffening of the extracellular matrix, mediated by oxidative remodeling, enhances mechanosensitive inflammatory signaling and perpetuates the advancement of fibrosis. As a result, oxidative stress is both a result of ovarian ageing and a significant enhancer of stromal degradation [59].

The antioxidant defence mechanisms diminish significantly with reproductive ageing, hence increasing vulnerability to oxidative damage. The diminished activity of superoxide dismutase, catalase, glutathione peroxidase, and other antioxidant enzymes limits the capacity of ovarian tissue to mitigate the overproduction of reactive oxygen species. Mitochondrial quality-control mechanisms, including mitophagy, progressively deteriorate, resulting in the buildup of defective mitochondria in ovarian cells [60]. Deficient removal of damaged mitochondria perpetuates chronic oxidative stress and induces continuous release of pro-inflammatory and pro-fibrotic signals into the adjacent stromal environment.

Oxidative stress contributes to DNA damage, telomere shortening, chromatin instability, and cellular senescence in many compartments of the ovary. Senescent granulosa cells and fibroblasts acquire a secretory phenotype marked by the persistent release of inflammatory cytokines, extracellular matrix proteins, growth factors, and reactive oxygen species, which may influence adjacent cells [61]. The accumulation of senescent cells establishes a detrimental loop connecting oxidative damage, persistent inflammation, extracellular matrix remodeling, and advancing tissue fibrosis. The interaction between senescent stromal cells and growing follicles may further diminish oocyte competence and expedite reproductive decline [62].

The mechanical modifications resulting from fibrosis might further intensify oxidative stress. The gradual hardening of the tissue alters the vascular structure, reduces oxygen transport, and facilitates the formation of hypoxic patches within the cortex. Hypoxia-induced mitochondrial dysfunction may subsequently elevate the generation of reactive oxygen species and enhance inflammatory signaling pathways. Consequently, biomechanical irregularities and oxidative stress are interrelated, creating a detrimental loop that may gradually disrupt ovarian homeostasis [63]. Beyond apoptosis and senescence, ferroptosis, an iron-dependent form of regulated cell death driven by uncontrolled lipid peroxidation, has emerged as a distinct contributor to granulosa cell loss in the ageing and fibrotic ovary. This pathway has been implicated in chemotherapy-induced ovarian insufficiency, polycystic ovary syndrome, and age-related granulosa cell death, operating alongside conventional apoptotic mechanisms. The SIRT1-Nrf2 axis functions as a principal safeguard against this process, sustaining expression of antioxidant and detoxifying enzymes such as GPX4, SOD2, and HO-1; disruption of this axis accelerates granulosa cell senescence and follicular depletion, linking ferroptotic vulnerability directly to the oxidative and fibrotic processes described above.

Growing experimental data underscores the therapeutic significance of oxidative stress regulation in ovarian fibrosis. Antioxidant drugs, mitochondrial stabilisers, anti-inflammatory medicines, and anti-fibrotic therapies have shown variable efficacy in diminishing collagen deposition and enhancing ovarian architecture in preclinical models [6]. Laboratory investigations have shown that metformin, melatonin, mitochondrial-targeted antioxidants, and extracellular matrix-modulating treatments may provide protective benefits against stromal remodeling, an area discussed in greater detail in Section 7.3. Although translational human data are limited, the increasing recognition that oxidative stress significantly exacerbates ovarian fibrosis may provide novel treatment avenues to maintain reproductive function and delay reproductive ageing. There is growing evidence that oxidative stress may serve as a crucial biological connection among mitochondrial failure, inflammation, extracellular matrix remodeling, cellular senescence, and fibrosis in the ageing ovary. Direct mechanical measurements illustrate the scale of this difference: instrumental indentation of mouse ovaries shows tissue stiffness rising from 1.98 ± 0.42 kPa in young animals to 4.36 ± 1.24 kPa in aged animals, and equivalent measurements in human ovarian tissue show a comparable shift, from 3.2 ± 0.25 kPa in reproductive-age women to 7.1 ± 0.71 kPa in menopausal women, providing a concrete biomechanical benchmark that distinguishes a compliant, reproductively active ovary from a stiffened, fibrotic one [22].

## 5. Mechanotransduction and Oocyte Competence

### 5.1. Hippo Signaling and Mechanical Regulation of Follicular Activation

The mechanical control of ovarian physiology has emerged as a rapidly expanding topic in reproductive biology in recent years. Traditional models of follicle formation have highlighted endocrine signaling, gonadotropin responsiveness, and local growth factor activity. The significance of tissue mechanics has been mostly overlooked [26]. Emerging experimental data indicates that ovarian cells continuously sense and respond to biomechanical pressures generated by extracellular matrix stiffness, tissue tension, cytoskeletal contraction, follicular expansion, and local pressure gradients within the ovarian cortex, through dedicated mechanosensitive machinery such as the Piezo1 ion channel, which converts mechanical stimuli into intracellular calcium signaling and, in ovarian cells, has been shown to directly activate the Hippo/YAP pathway discussed below. The physical characteristics of the ovarian microenvironment seem to influence primordial follicle activation, granulosa cell proliferation, metabolic adaption, and oocyte competence without reliance on traditional hormonal mechanisms. The acknowledgement of these biomechanical impacts has significantly enhanced the understanding of the processes that preserve ovarian homeostasis throughout reproductive life [64].

Primordial follicles are mostly located in the dense cortical region of the ovary, which is characterized by high collagen content, increased rigidity, and reduced vascularization. This mechanically limited environment must be maintained to maintain follicular dormancy and avoid excessive recruitment of the primordial follicle reserve [65]. Consequently, incremental alterations in cortical architecture may significantly affect the equilibrium between follicular quiescence and activity. Growing experimental data suggests that biomechanical cues from the ovarian cortex are crucial regulators of follicular dynamics. Excessive tissue rigidity may impose mechanical limitations on follicular development, whereas loss of cortical integrity might initiate aberrant follicular recruitment and expedite the depletion of the ovarian reserve [66].

It is useful to distinguish two mechanistically and temporally distinct tiers of this cascade: an immediate mechanosensitive response, in which channels such as Piezo1 transduce mechanical stimuli into rapid intracellular calcium signaling within seconds, and a slower, downstream transcriptional response, in which the Hippo pathway integrates this and other cytoskeletal cues to regulate the nuclear localization of the transcriptional coactivators YAP and TAZ over a gene-expression-dependent timescale. The remainder of this section focuses on this second, transcriptional tier.

The Hippo signaling system has recently been identified as a crucial biochemical mechanism linking tissue mechanics and ovarian physiology. The Hippo pathway, first recognized as a modulator of organ size and cell proliferation, is now shown to be extremely responsive to extracellular matrix composition, actin cytoskeleton arrangement, cellular polarity, and mechanical strain [67]. Physiologically, the activation of Hippo kinase upstream restricts the nuclear translocation of the transcriptional coactivators YAP and TAZ, hence controlling cellular proliferation and tissue homeostasis. Mechanical modifications affecting cytoskeletal structure may impede Hippo signaling and facilitate the activation of downstream proliferative pathways. This biomechanical regulation is particularly significant in the ovary, where precise coordination of follicular recruitment is essential for sustained reproductive activity [68].

Significant data substantiating the correlation between ovarian biomechanics and follicle activation comes from the experimental fragmentation of ovarian tissue. Mechanical disturbance of cortical architecture induces actin polymerization and alters intracellular tension, eventually inhibiting Hippo signaling and facilitating the nuclear translocation of YAP [26]. The subsequent activation of YAP-dependent transcriptional pathways facilitates the development of latent follicles. These results conclusively indicated that follicular recruitment may be directly affected by mechanical manipulation of ovarian tissue, independent of endocrine stimulation. The advancement of in vitro activation techniques using ovarian fragmentation underscored the translational importance of mechanotransduction pathways in reproductive medicine [69].

Granulosa cells seem to be very responsive to alterations in mechanical signaling. Changes in the stiffness of the extracellular matrix affect cytoskeletal organization, focal adhesion assembly, intracellular tension, and various downstream transcriptional pathways that govern proliferation, differentiation, apoptosis resistance, and metabolic activity. YAP and TAZ interact with signaling networks associated with oxidative stress response, mitochondrial function, glucose metabolism, and inflammatory control [27]. Thus, mechanical dysregulation of Hippo signaling may result in both the inhibition of primordial follicle activation and the compromise of the functional integrity of the developing follicular unit. Chronic exposure to aberrant biomechanical circumstances may gradually undermine follicular homeostasis and reproductive competence [70,71,72].

### 5.2. Extracellular Matrix Stiffness and Follicular Development

The mechanical qualities of the extracellular matrix are essential for the development, maturity, and survival of the follicle. Folliculogenesis involves continuous alteration of the surrounding stromal environment to support fast follicular growth, granulosa cell proliferation, vascular adjustments, and heightened metabolic requirements [13]. The typical pliability of the extracellular matrix enables follicles to enlarge while preserving structural integrity and facilitating coordinated cellular communication. As the ovarian stroma becomes more rigid over time, it can create an environment that is too mechanically restrictive to allow for normal follicular growth. There is growing evidence that the stiffness of the extracellular matrix may independently regulate reproductive competence [9].

Collagen and the cross-linking of the extracellular matrix increasingly accumulate throughout reproductive ageing and fibrotic remodeling, resulting in increased ovarian stiffness. Excessive deposition of collagen fibres modifies the local tissue tension and the biomechanical environment around growing follicles. Dense collagen networks may physically impede follicular development, hinder antral cavity formation, and disturb the typical spatial arrangement of granulosa cells around the oocyte. Furthermore, heightened stiffness of the extracellular matrix may physically constrict the follicular milieu, hindering the flow of nutrients, hormones, and oxygen. Such significant structural aberrations represent considerable changes in the context of follicular development [3].

Irregular tissue biomechanics may substantially influence follicular fluid dynamics. Progressive stromal stiffness modifies local pressure gradients and may disrupt the physiological expansion of the follicular antrum [73]. The mechanical constraints of an expanding follicle may impede oxygen transport, nutrient diffusion, and the removal of metabolic waste inside the follicular compartment [3]. Disruption of these microenvironmental variables may progressively disrupt cellular homeostasis and heighten vulnerability to oxidative stress and inflammatory damage. Cumulative exposure to extended, mechanically abnormal situations may result in diminished reproductive function [74].

The stiffness of the extracellular matrix also affects vascular architecture in the ovary. Excessive collagen accumulation may constrict microvascular structures, hindering tissue perfusion and diminishing angiogenic efficacy. The hypoxic microenvironments that develop may exacerbate oxidative stress, inflammatory signaling, and mitochondrial dysfunction in both stromal and follicular compartments [63]. A decrease in oxygen availability may further hinder granulosa-cell metabolism and disrupt energy-dependent activities essential for oocyte maturation. A crucial component of the progressive decline in ovarian function is the relationship between extracellular matrix stiffening and vascular impairment [75,76,77].

The increasing interest in ovarian biomechanics has led to the creation of novel diagnostic methods aimed at in vivo evaluation of ovarian stiffness. Initial elastographic investigations indicate a correlation between heightened ovarian stiffness and reproductive ageing, polycystic ovary syndrome, and reduced ovarian reserve [78]. The biomechanical characterization of ovarian tissue may serve as a therapeutically significant biomarker that indicates biological ovarian quality beyond traditional endocrine markers. Possible benefits of such methods include more individualized reproductive assessment and a deeper understanding of ovarian ageing.

### 5.3. Mechanical Regulation of Granulosa–Oocyte Communication

One of the key factors influencing oocyte quality and developmental competency is bidirectional communication between granulosa cells and the oocyte. Folliculogenesis requires the ongoing flow of metabolites, amino acids, lipids, ions, growth factors, signaling molecules, and energy substrates to sustain meiotic arrest, facilitate cytoplasmic maturation, maintain mitochondrial function, and coordinate cellular metabolism [79]. The operational integrity of this communication network relies not only on endocrine control but also on the maintenance of an appropriate mechanical environment inside the follicular niche. An increasing amount of data suggests that biophysical changes in the ovarian microenvironment may significantly affect granulosa–oocyte communication and reproductive competence [80].

Gap junctions between granulosa cells and oocytes exhibit heightened sensitivity to cytoskeletal arrangement and intracellular stress. Mechanical stress resulting from extracellular matrix stiffening may disrupt intercellular communication pathways and inhibit the delivery of essential metabolites and signaling molecules into the oocyte. Oocytes possess a limited glycolytic ability and rely significantly on adjacent granulosa cells for energy substrates, redox control, and metabolic assistance. The disruption of this metabolic collaboration may therefore lead to impaired ATP synthesis, mitochondrial homeostasis, calcium signaling, and meiotic control in the developing oocyte. The mechanical dysregulation of granulosa–oocyte connection may limit oocyte competency, even in physically normal follicles [81]. Consistent with this, connexin 43 expression and gap-junctional coupling measured directly in human cumulus cells from IVF/ICSI patients correlate positively with embryo quality and pregnancy outcome, providing rare direct human evidence for this pathway [82].

The gap junctions described above are physically housed within transzonal projections (TZPs), thin actin- and microtubule-based cytoplasmic extensions that granulosa cells send through the zona pellucida to directly contact the oocyte membrane, forming the only structural route for contact-dependent communication between the two cell types. Because TZPs depend on a functional cytoskeletal network to form, extend, and persist, they are inherently mechanosensitive structures, and their density declines with disruption of the surrounding actin or microtubule architecture. This vulnerability has direct clinical relevance: in polycystic ovary syndrome, TZP density is reduced and calcium/calmodulin-dependent kinase II beta signaling is dysregulated in both patient samples and animal models, directly linking TZP disruption to impaired oocyte–granulosa communication in a fibro-inflammatory, hyperandrogenic ovarian environment.

Specifically, it seems that mitochondrial activity in granulosa cells is quite vulnerable to aberrant biomechanical state. Cytoskeletal tension governs mitochondrial localization, membrane potential, oxidative phosphorylation efficiency, and reactive oxygen species generation [82]. Consequently, mechanical stress resulting from stromal fibrosis may produce significant metabolic alterations in granulosa cells, subsequently impacting the oocyte. Dysfunctional granulosa cells may lead to diminished metabolic support, affecting substrate availability, antioxidant protection, and energy coordination essential for optimal oocyte maturation. Consequently, increasing biomechanical degradation of the ovarian stroma may indirectly compromise reproductive capability by disrupting somatic cell metabolism [83].

Mechanical signaling also influences the intracellular calcium dynamics of granulosa cells and oocytes. Mechanosensitive ion channels react to extracellular matrix stress and control calcium influx, cytoskeletal remodeling, and subsequent transcriptional pathways that govern follicular development [84]. Disruption of calcium signaling may impair meiotic progression, spindle organization, and cytoplasmic maturation processes essential for proper fertilization and embryonic development. This indicates that prolonged exposure to atypical mechanical conditions may gradually destabilize various intracellular systems essential for the preservation of oocyte quality.

The cytoskeletal architecture inside the oocyte may be disrupted under situations of severe biomechanical stress. Microtubules are structural components of the meiotic spindle that must be tightly regulated and assembled with great coordination in order to ensure proper orientation. Increased oxidative stress, alterations in mitochondrial function, and disturbances in calcium signaling and metabolic instability resulting from extracellular matrix stiffening may jeopardize spindle integrity and heighten susceptibility to chromosomal segregation mistakes. It is well established that ovarian fibrosis, diminishing embryonic competence, and reproductive ageing are associated, and these processes may play a role in this association [85].

Mechanical alterations also affect paracrine signaling pathways within the follicular milieu. Granulosa cells synthesize growth factors, cytokines, extracellular vesicles, and metabolic mediators that continuously control oocyte maturation and developmental competence [86]. Variations in the rigidity of the extracellular matrix may affect secretion patterns and modify the responsiveness of granulosa cells to hormonal and metabolic stimuli. Folliculogenesis relies on coordinated communication networks, which may be disturbed during fibrotic remodeling, which in turn changes the molecular signaling landscape of the follicular niche [4].

Table 1 delineates the principal pathways implicated in ovarian fibro-inflammatory reconstruction, encompassing stromal fibrosis, extracellular matrix dysregulation, inflammation, oxidative stress, and mechanotransduction anomalies. It should be noted that, with the exception of gap-junctional signaling, most of the mechanistic evidence presented in this section, particularly regarding calcium dynamics and meiotic spindle regulation, derives from in vitro systems or mouse models, and direct confirmation in human oocytes remains largely unestablished. A comprehensive overview of the key molecular pathways, stromal modifications, inflammatory mediators, and mechanotransduction mechanisms involved in ovarian fibrosis and reproductive dysfunction related to reproductive ageing, PCOS, diminished ovarian reserve, obesity-related infertility, and endometriosis.

## 6. Ovarian Fibrosis in Female Infertility Disorders

The four conditions discussed in this section share two recurring mechanisms, described once here rather than separately in each subsection. First, collagen and extracellular matrix accumulation raises ovarian stromal stiffness, and the resulting change in tissue tension around growing follicles disrupts integrin-mediated and Hippo/YAP-TAZ mechanotransduction, impairing follicular activation and granulosa-cell function. Second, this same stiffening compresses the ovarian microvasculature, producing local hypoxia that activates HIF-1α signaling and further drives inflammatory and pro-fibrotic pathways, compounding oxidative stress and impairing granulosa-cell and oocyte metabolism [3]. Each subsection below notes only how these two mechanisms manifest in that specific condition.

### 6.1. Polycystic Ovary Syndrome (PCOS) and Stromal Fibrosis

Polycystic ovary syndrome is one of the most prevalent endocrine and metabolic illnesses linked to female infertility, increasingly recognized as a condition characterized by significant stromal remodeling and fibro-inflammatory ovarian dysfunction. Conventional classical morphological characterizations of polycystic ovaries often include augmented ovarian volume, stromal hypertrophy, cortical thickness, hypervascularity, and the accumulation of many tiny antral follicles that are developmentally stalled [92]. Recent molecular and histopathological evidence indicates that these structural abnormalities are more likely indicative of significant dysregulation in extracellular matrix organization, inflammatory signaling, mechanotransduction pathways, and stromal cell activity, rather than merely the result of chronic anovulation. The progressive fibrotic remodeling of the ovarian milieu may serve as an active pathogenic factor in PCOS, directly influencing follicular dynamics and reproductive capability [93].

It is important to note that PCOS is not a uniform entity with respect to fibro-inflammatory burden. Under the Rotterdam classification, phenotypes A and B, which combine hyperandrogenism with anovulation, carry the highest metabolic and inflammatory risk, including insulin resistance and elevated markers of low-grade inflammation; phenotype C, though ovulatory, still shows metabolic abnormalities driven by androgen excess; while phenotype D, lacking hyperandrogenism altogether, generally presents a milder hormonal and inflammatory profile. Much of the stromal fibrosis and inflammatory signaling discussed below has been studied predominantly in hyperandrogenic phenotypes, and the extent to which it extends to phenotype D remains comparatively unestablished.

Polycystic ovaries exhibit stromal hyperplasia accompanied by notable changes in extracellular matrix composition and collagen arrangement. Histological examination reveals elevated deposition of collagen types I and III, expedited cross-linking of the extracellular matrix, and enlargement of fibrotic stromal compartments in the cortical and medullary regions of the ovary. Excessive accumulation of fibronectin, laminins, and matrix-associated glycoproteins results in alterations to tissue architecture and heightened stromal stiffness [94]. The concurrent imbalance of matrix metalloproteinases and tissue inhibitors of metalloproteinases disturbs normal extracellular matrix turnover, leading to increased collagen buildup. Many steps of normal folliculogenesis may be disrupted as a result of these alterations, which have a major effect on the biomechanical environment of growing follicles [95].

Hyperandrogenism seems to be a primary molecular factor in stromal remodeling in PCOS. Research has demonstrated that prolonged exposure to androgens activates fibroblasts, enhances collagen production, and increases the expression of pro-fibrotic genes, such as TGF-β, connective tissue growth factor, α-smooth muscle actin, and collagen transcription regulators. Furthermore, the activation of androgen receptors in stromal cells regulates pathways associated with the remodeling of the extracellular matrix via interactions with the PI3K/AKT, MAPK, and SMAD signaling pathways. As a result, chronic androgen surplus may contribute to sustained fibroblast activation and increasing extracellular matrix formation in the ovarian stroma [96].

Granulosa-cell dysfunction produced by hyperandrogenism exacerbates fibro-inflammatory remodeling. Excess androgen exposure impedes granulosa cell differentiation, diminishes aromatase activity, modifies FSH response, and obstructs proper follicular maturation. Follicles arrested in PCOS often exhibit aberrant proliferation of granulosa cells, mitochondrial dysfunction, oxidative stress buildup, and altered production of extracellular matrix-modulating cytokines. The disruption of granulosa–stromal communication may contribute to the gradual remodeling of the ovarian milieu and facilitate the maintenance of follicular arrest [97].

A significant factor in fibrotic remodeling in PCOS is persistent low-grade inflammation. Elevated circulating and follicular fluid concentrations of tumour necrosis factor-α, interleukin-1β, interleukin-6, monocyte chemoattractant protein-1, TGF-β, and C-reactive protein have been frequently seen in women with PCOS [87]. Inflammatory signaling pathways, including nuclear factor-kappa B, JNK, STAT3, and inflammasome-related mediators, induce persistent stromal damage and fibroblast activation. Extended inflammatory activation may adversely affect matrix breakdown mechanisms and facilitate the buildup of rigid extracellular matrix forms that resist physiological remodeling.

Macrophage infiltration in polycystic ovaries is intimately linked to the onset of persistent stromal inflammation and fibrosis. Macrophage polarization shifts promote the prevalence of pro-inflammatory phenotypes, resulting in heightened synthesis of cytokines, reactive oxygen species, and pro-fibrotic growth factors. Interactions between activated macrophages and stromal fibroblasts enhance extracellular matrix accumulation and perpetuate chronic inflammatory signaling inside the ovarian niche. Macrophage-driven mechanisms that induce fibro-inflammatory responses have been associated with systemic fibrotic diseases and are becoming acknowledged as pertinent to ovarian pathology in PCOS [89].

This results in heightened fibro-inflammatory signaling in the ovary due to insulin resistance and hyperinsulinemia. Hyperinsulinemia enhances androgen production in theca cells and activates inflammatory and pro-fibrotic pathways via PI3K/AKT/mTOR and MAPK signaling. Metabolic imbalance results in mitochondrial malfunction, excessive generation of reactive oxygen species, and oxidative damage in both stromal and follicular compartments [98]. Elevated free fatty acid levels and adipokine dysregulation associated with obesity may exacerbate inflammatory activation and extracellular matrix remodeling. Metabolic dysregulation, chronic inflammation, androgen excess, and oxidative stress all work together to form a pathological network that may sustain ovarian fibrosis progression.

In PCOS, this shared mechanism appears to help sustain the follicular quiescence characteristic of the condition, with the compression-driven hypoxia further amplified by the insulin resistance and hyperinsulinemia already discussed above [25,99].

Additional data from elastographic imaging investigations indicates that ovarian biomechanics are modified in women with PCOS. Numerous studies indicate heightened ovarian stiffness relative to healthy controls, suggesting that stromal remodelling may be quantifiable in vivo. The biomechanical characterisation of ovarian tissue may evolve into a clinically significant indicator for disease severity, inflammatory activity, or reproductive potential in women with PCOS. These methodologies may eventually complement existing assessment techniques reliant on endocrinological and ultrasonographic evaluations [100].

Fibrotic remodelling may potentially contribute to the variability in reproductive results seen in women with PCOS undergoing assisted reproduction. While ovarian stimulation often yields a substantial quantity of oocytes, alterations in extracellular matrix architecture, oxidative stress pathways, inflammatory signalling, and granulosa–oocyte communication may impact the developmental competency of the harvested oocytes. Accordingly, mechanical and metabolic irregularities within the follicular milieu may partially explain the diminished embryo quality and inconsistent implantation potential seen in some PCOS individuals, despite seemingly adequate ovarian reserve metrics [80]. The principal fibro-inflammatory characteristics linked to prevalent infertility-related diseases are encapsulated in Table 2.

Comparison of the major fibro-inflammatory, extracellular matrix, oxidative, vascular, and biomechanical abnormalities identified across common infertility-related ovarian disorders.

### 6.2. Diminished Ovarian Reserve and Premature Ovarian Insufficiency

Diminished ovarian reserve (DOR) and premature ovarian insufficiency (POI) are reproductive disorders marked by a rapid drop in ovarian function, reduced follicular responsiveness, and a gradual loss of reproductive capability. The traditional theories attribute these illnesses primarily to a quantitative reduction in the primordial follicular reserve [102]. It is worth distinguishing between the major etiological categories of POI, as their fibro-inflammatory contributions differ considerably. In a recent cohort, iatrogenic causes, chiefly chemotherapy, radiotherapy, and ovarian surgery, accounted for 34.2% of cases, autoimmune causes 18.9%, and genetic causes 9.9%, while the remaining 36.9% were classified as idiopathic [102]. Iatrogenic POI typically follows an acute insult with direct follicular depletion and secondary stromal scarring, autoimmune POI is driven by primary lymphocytic infiltration and antibody-mediated damage to the stroma and steroidogenic cells, whereas idiopathic POI most likely represents a heterogeneous mixture of undetected genetic and subclinical inflammatory mechanisms rather than a single fibro-inflammatory pathway. There is growing data that underscores the significance of the ovarian microenvironment, with its degeneration potentially playing a crucial, independent role in follicular dysfunction and reproductive ageing, irrespective of follicle quantity alone. Fibro-inflammatory remodelling, extracellular matrix buildup, vascular impairment, mitochondrial dysfunction, and persistent stromal damage are now closely associated with the pathogenesis of both DOR and POI [90].

Histological analysis of ovarian tissue from women with low ovarian reserve often demonstrates extensive stromal disorganisation, increased collagen deposition, extracellular matrix remodelling, decreased vascular density, and chronic inflammatory activation. Consistent analogous alterations were noted in experimental ageing models characterised by diminished fertility and compromised folliculogenesis. The gradual substitution of normal stromal architecture with fibrotic tissue may provide a mechanically constrictive environment that is incapable of facilitating physiological follicular activation and development. This extent of structural degeneration may jeopardise ovarian function prior to the clinical identification of residual follicle depletion [106].

The persistent stimulation of TGF-β signalling seems to significantly contribute to stromal fibrosis associated with DOR and POI. Elevated TGF-β activity enhances fibroblast proliferation, extracellular matrix formation, and collagen cross-linking, and suppresses matrix breakdown mechanisms. Concurrently, SMAD-dependent transcriptional pathways govern the production of fibrotic mediators, including connective tissue growth factor, fibronectin, α-smooth muscle actin, and numerous collagen-associated proteins. Ovarian stroma undergoes a gradual remodelling into a stiff fibro-inflammatory milieu due to the chronic activation of these pathways; this remodelling is linked to diminished reproductive competence and poor follicular support [107].

Oxidative stress and mitochondrial dysfunction contribute to stromal remodelling in reduced ovarian reserve and premature ovarian insufficiency. Age-related deterioration of mitochondrial oxidative phosphorylation leads to heightened production of reactive oxygen species, resulting in oxidative damage to granulosa cells, stromal fibroblasts, endothelial cells, and remaining follicles [108]. Excessive oxidative stress triggers inflammatory signalling pathways regulated by NF-κB, p38 MAPK, and inflammasome-related mediators, while also promoting extracellular matrix formation and fibroblast proliferation. So, it is possible that oxidative stress, fibrosis, and mitochondrial dysfunction all work together to hasten ovarian decline.

Cellular senescence seems to be central to the pathogenesis of ovarian fibrosis in DOR and POI. Senescent stromal fibroblasts have a secretory phenotype characterised by elevated production of inflammatory cytokines, growth factors, extracellular matrix proteins, and reactive oxygen species. Granulosa-cell senescence may also hinder steroidogenesis, mitochondrial metabolism, and intercellular communication between granulosa cells and oocytes. Consequently, the interaction between senescent stromal cells and adjacent follicles may create a persistently inflammatory and physiologically unstable ovarian milieu detrimental to follicular survival and development [86].

In DOR and POI, both mechanisms act on an already depleted follicle pool: the residual follicles are exposed to abnormal mechanical stress and hypoxic compromise that may undermine their developmental competence independent of chronological age [3,109].

Immune dysregulation seems to be especially pertinent in some types of premature ovarian insufficiency. Autoimmune-associated primary ovarian insufficiency is often marked by significant inflammatory infiltration, cytokine activation, and stromal remodelling in ovarian tissue. Nonetheless, sustained immune stimulation may induce fibroblast activation and extracellular matrix buildup, leading to direct follicular injury. Comparable fibro-inflammatory pathways may lead to ovarian impairment after chemotherapy, radiation exposure, or significant metabolic stress [110]. In line with this, metabolomic profiling in a cyclophosphamide-induced model of premature ovarian failure has identified specific metabolic disturbances associated with chemotherapy-related follicular loss, supporting a shared metabolic dimension to chemotherapy-induced and fibrosis-associated ovarian insufficiency [111].

Data from assisted reproduction indicates the increased biological significance of stromal remodeling in reduced ovarian reserve. Women with DOR or POI, although possessing some residual follicles, often exhibit compromised ovarian response, diminished oocyte competence, mitochondrial malfunction, and decreased embryonic developmental potential. The structural deterioration of the ovarian microenvironment may directly contribute to deteriorating reproductive results, beyond the simple loss of follicle quantity. Ovarian insufficiency is now seen from a broader angle thanks to the discovery that fibro-inflammatory remodeling is inherent to DOR and POI. Reproductive decline is linked to a gradual destabilization of the ovarian ecosystem, including oxidative stress, mitochondrial failure, chronic inflammation, extracellular matrix remodeling, vascular impairment, and biomechanical dysregulation. In turn, forthcoming treatments for safeguarding reproductive lifespan and enhancing fertility outcomes may include the preservation of stromal homeostasis [13].

### 6.3. Endometriosis-Associated Ovarian Fibrosis and Microenvironmental Remodeling

Endometriosis is a complicated inflammatory disorder linked to female infertility and is becoming recognized as a contributor to significant fibro-inflammatory alteration in the ovarian microenvironment. Ovarian endometriosis, especially as endometriomas, exposes ovarian tissue to persistent inflammation, oxidative stress, recurrent hemorrhagic damage, and ongoing extracellular matrix remodeling [103]. The fibrotic impact of endometriosis on the ovary differs depending on lesion type. Endometriomas cause direct structural damage to the ovarian cortex through cyst wall invasion and repeated intracystic hemorrhage, whereas deep infiltrating endometriosis (DIE) typically develops in extra-ovarian sites such as the rectovaginal septum and uterosacral ligaments, and its effect on ovarian reserve is thought to arise more from shared pelvic inflammation, adhesion formation, and the extent of associated surgery than from direct infiltration of ovarian tissue. Histologically, however, the two lesion types show comparable fibrotic transformation, with deep infiltrating lesions displaying even greater fibroblast-to-myofibroblast transdifferentiation, smooth muscle metaplasia, and fibrotic content than endometriomas, alongside reduced vascularity, indicating that the fibrotic process itself is not confined to the ovary but is a shared feature of the disease across anatomical sites [103]. The structural damage linked to endometriotic lesions surpasses the apparent cystic chamber and often encompasses significant alterations in the adjacent ovarian cortex and stromal compartments. As a result, increasing fibrosis and the disruption of normal ovarian architecture may directly lead to diminished ovarian reserve, poor folliculogenesis, and decreased reproductive competence in afflicted individuals [104].

Recurrent cyclic haemorrhage into endometriotic lesions creates a markedly inflammatory and oxidative intrafollicular milieu characterized by the buildup of haemoglobin breakdown products, free iron, reactive oxygen species, and lipid peroxidation metabolites. Iron overload, caused by repeated haemorrhaging, seems to be significant in promoting oxidative tissue injury and fibroblast activation. Excess iron accumulation promotes the generation of reactive oxygen species via Fenton chemistry, aggravating mitochondrial dysfunction, inflammatory signaling, and extracellular matrix remodeling in adjacent ovarian tissue. Fibrotic cortical scarring and stromal integrity impairment are both brought about by prolonged oxidative stress, which is itself caused by these processes [112].

TGF-β signaling has a crucial role in the progression of fibrosis in ovarian endometriosis. Elevated TGF-β levels in endometriotic lesions stimulate fibroblast proliferation, extracellular matrix formation, collagen cross-linking, and myofibroblast differentiation [113]. Continuous activation of SMAD-dependent transcriptional pathways enhances the production of fibrotic mediators, including collagen type I, fibronectin, connective tissue growth factor, and α-smooth muscle actin. Simultaneously, the inhibition of matrix breakdown pathways facilitates the gradual buildup of extracellular matrix and the stiffening of stromal tissue. Prolonged stimulation of these pathways may significantly influence ovarian biomechanics and impact follicular growth in the surrounding tissue [114].

The activation of myofibroblasts is a key histological characteristic of fibrosis linked to endometriosis. Chronic inflammatory and oxidative circumstances lead to the gradual development of contractile and extracellular matrix-producing phenotypes in stromal fibroblasts, marked by the expression of α-smooth muscle actin and increased collagen production. Activated myofibroblasts contribute to the buildup of extracellular matrix, tissue contraction, vascular distortion, and the eventual disruption of normal ovarian architecture. The continued presence of activated myofibroblasts in ovarian tissue may sustain chronic fibrosis even with the surgical removal of endometriotic lesions [6].

Inflammatory signalling in ovarian tissue associated with endometriosis entails significant activation of macrophages, neutrophils, mast cells, and pro-inflammatory cytokine networks. Tumour necrosis factor-α, interleukin-1β, interleukin-6, prostaglandins, chemokines, and inflammasome-associated mediators are consistently higher in women diagnosed with endometriosis. The activation of the NF-κB, STAT3, MAPK, and inflammasome pathways exacerbates chronic inflammatory remodeling and promotes extracellular matrix deposition. Chronic inflammation also diminishes granulosa cell functionality and may interfere with communication between somatic cells and the oocyte [88].

Oxidative stress seems to be fundamentally connected to inflammatory and fibrotic processes in ovarian endometriosis. Excessive production of reactive oxygen species damages mitochondrial DNA, proteins, lipids, and cytoskeletal structures in granulosa cells and adjacent stromal compartments [81]. Mitochondrial failure resulting from oxidative stress may impair ATP generation, calcium signaling, and metabolic support to the oocyte. Concurrently, oxidative stress initiates profibrotic signaling pathways and expedites extracellular matrix remodeling. The persistent interaction between oxidative damage and fibrosis is crucial to the deterioration of ovarian function in endometriosis [115].

In endometriosis, both mechanisms are compounded by the iron- and TGF-β-driven inflammatory milieu of the lesions themselves, adding to the self-sustaining cycle of hypoxia, inflammation, oxidative stress, and fibrosis already described in the surrounding ovarian cortex [28,91].

The surgical therapy of ovarian endometriomas may contribute to further stromal damage and fibrotic advancement. Cystectomy may ease pain sensations and facilitate access to follicles for assisted reproduction. Nevertheless, the removal of endometriotic tissue may inadvertently damage adjacent healthy ovarian cortex and vascular systems. Post-surgical tissue healing mechanisms, including inflammatory activation and extracellular matrix remodeling, may further aggravate stromal fibrosis and hasten the deterioration of ovarian reserve. Finding a balance between disease management and preserving ovarian function may be challenging for women with endometriosis, as these data demonstrate [7].

Investigations of follicular fluid reveal significant molecular alterations in ovaries affected by endometriosis. Follicles from these women have elevated amounts of inflammatory cytokines, indicators of oxidative stress, iron-related metabolites, and extracellular vesicles containing inflammatory components. Granulosa cells may demonstrate mitochondrial dysfunction, modified steroidogenesis, compromised antioxidant defences, and heightened apoptotic activity when exposed to this diseased microenvironment. Such alterations may directly contribute to diminished oocyte competence and reduced embryonic developmental potential in assisted reproduction [116].

Clinical reproductive results further substantiate the biological significance of ovarian fibrosis in endometriosis. Women with ovarian endometriosis often exhibit diminished ovarian reserve, reduced ovarian response, lower oocyte yield, compromised embryo quality, and decreased implantation potential after IVF therapy [117]. Consequently, the structural degradation of the ovarian microenvironment may significantly influence reproductive decline, beyond the mechanical distortion induced by the endometrioma itself [118].

### 6.4. Obesity, Metabolic Dysfunction, and Ovarian Fibrosis

Obesity is a significant metabolic disease that contributes to female infertility and adverse reproductive consequences globally. In addition to its endocrine and systemic metabolic consequences, there is increasing evidence of obesity-induced extensive modification of the ovarian microenvironment via chronic inflammation, oxidative stress, lipotoxicity, mitochondrial dysfunction, and the gradual buildup of the extracellular matrix [105].

The magnitude of this effect appears to follow a dose–response pattern with increasing body mass index: compared with women of normal weight, those with a BMI of 30 kg/m^2^ or higher undergoing IVF show significantly lower clinical pregnancy and live birth rates, with declining outcomes as BMI rises further [105]. Fat distribution, and not BMI alone, also matters: women with comparable BMI but greater visceral and central adiposity, as reflected by waist circumference, show higher fasting insulin, greater insulin resistance, and poorer ovarian stimulation and oocyte yield than those with a predominantly subcutaneous distribution [105]. This distinction is directly relevant to local ovarian fibrosis, since the fat depot immediately surrounding the ovary behaves as a visceral, rather than subcutaneous, adipose compartment: histological analysis of this peri-ovarian fat shows that it undergoes its own structural and lipid remodeling that parallels, and may directly contribute to, the fibro-inflammatory changes occurring in the adjacent ovarian stroma [105].

Ovarian dysfunction associated with obesity cannot be exclusively ascribed to alterations in hypothalamic–pituitary–ovarian transmission or insulin resistance. The structural and biomechanical degradation of the ovarian stroma seems to directly contribute to follicular dysfunction and reduced oocyte competency in metabolically impaired individuals [119].

In obesity, adipose tissue operates as a persistently inflamed endocrine organ capable of profoundly influencing ovarian physiology. Hypertrophic adipocytes release elevated amounts of tumour necrosis factor-α, interleukin-6, leptin, resistin, monocyte chemoattractant protein-1, and several pro-inflammatory adipokines, resulting in systemic and localized inflammatory activation [120]. Prolonged exposure of ovarian tissue to an inflammatory environment promotes macrophage infiltration, fibroblast activation, extracellular matrix deposition, and oxidative damage in the ovarian stroma. As a result, prolonged low-grade inflammation creates a fibro-inflammatory environment that may gradually alter ovarian architecture and follicular equilibrium.

Insulin resistance is a significant contributor to ovarian remodeling in obesity. Hyperinsulinemia stimulates the PI3K/AKT/mTOR and MAPK signaling pathways in ovarian tissue, resulting in alterations in granulosa cell metabolism, steroidogenesis, and follicular maturation. The overactivation of insulin signaling promotes inflammatory and pro-fibrotic communication cascades via interactions with TGF-β, nuclear factor-kappa B, and oxidative stress signaling pathways. Chronic insulin resistance leads to metabolic dysregulation, which may directly cause extracellular matrix buildup and advancing stromal fibrosis [121].

Lipotoxicity exacerbates ovarian damage in obesity-associated infertility. Elevated circulating free fatty acids and ectopic lipid accumulation in ovarian tissue result in mitochondrial dysfunction, endoplasmic reticulum stress, and heightened reactive oxygen species generation [122]. Lipotoxicity impacts mitochondrial membrane potential, ATP synthesis, and steroidogenic function, and heightens the vulnerability of granulosa cells to apoptosis. Concurrently, oxidative stress induced by lipid excess activates fibroblast growth pathways and extracellular matrix remodeling. As a result, prolonged exposure to lipotoxic conditions may gradually undermine the stability of both the stromal and follicular compartments of the ovary [123].

Mitochondrial failure significantly contributes to the relationship between metabolic stress and ovarian fibrosis. Chronic inflammatory activation and excessive food intake diminish the efficacy of oxidative phosphorylation, leading to increased electron leakage from the mitochondrial respiratory chain and resulting in persistent generation of reactive oxygen species. Oxidative damage triggers inflammatory and pro-fibrotic signal pathways, including NF-κB, TGF-β, SMAD proteins, and inflammasome-related mediators. Mitochondrial malfunction may impair mitophagy and other cellular quality-control mechanisms, resulting in the buildup of damaged mitochondria that may induce persistent oxidative stress in ovarian tissue [124].

Fibroblast activation triggered by metabolic failure significantly contributes to extracellular matrix remodeling in obesity. Exposure to inflammatory cytokines, reactive oxygen species, hyperinsulinemia, and adipokines promotes collagen production, fibronectin deposition, and myofibroblast development in the ovarian stroma. The disrupted equilibrium between matrix metalloproteinases and tissue inhibitors of metalloproteinases, together with the buildup of inflexible collagen networks, exacerbates the dysfunction in normal extracellular matrix turnover. These methods may induce progressive stromal fibrosis, significantly impacting ovarian biomechanics and hindering follicular growth [25].

An imbalance in adipokines significantly influences ovarian mechanobiology and follicular signaling. Elevated leptin levels, often seen in obese patients, may diminish the sensitivity of granulosa cells, alter steroidogenesis, and provoke an inflammatory response within the follicular milieu. Decreased adiponectin activity significantly exacerbates insulin resistance, oxidative stress, and endothelial dysfunction. The interaction between adipokine communication and mechanotransduction pathways, including Hippo/YAP-TAZ, focal adhesion kinases, and integrin-mediated communication, may contribute to ovarian stiffness and extracellular matrix reconfiguration in metabolically challenged women [125].

In obesity, both mechanisms operate largely independently of the endocrine and metabolic pathways discussed above, adding a distinct mechanical and vascular contribution to follicular and granulosa-cell dysfunction [3,126].

Research on follicular fluid reveals significant molecular alterations in the ovarian microenvironment of obese individuals. Women with obesity undergoing assisted reproduction have elevated levels of inflammatory cytokines, oxidative stress indicators, free fatty acids, advanced glycation end products, and extracellular vesicles containing inflammatory components [127]. Granulosa cells in this physiologically adverse environment often exhibit mitochondrial dysfunction, heightened apoptotic signaling, modified glucose metabolism, and weakened antioxidant defences. These anomalies may directly lead to diminished oocyte competence, compromised embryo development, and decreased implantation potential. The clinical reproductive results provide robust evidence for the biological significance of ovarian fibro-inflammatory remodeling in obesity [128]. In IVF therapy, obese women often exhibit diminished ovarian response, changed oocyte morphology, worse embryo quality, decreased implantation rates, and an elevated risk of miscarriage. This suggests that endocrine problems are not the only factor in reproductive failure; the structural and metabolic degradation of the ovarian microenvironment also plays a significant role [105].

### 6.5. Comparative Synthesis Across Conditions

Taken together, the sections above indicate that ovarian fibrosis is not a single, uniform entity even within one diagnosis: PCOS phenotypes A/B, iatrogenic or autoimmune POI, ovarian endometrioma versus deep infiltrating endometriosis, and high-BMI or visceral-adiposity obesity phenotypes each carry a distinct fibro-inflammatory burden and, in several cases, a distinct dominant mechanism, such as androgen-driven fibroblast activation in PCOS, primary lymphocytic damage in autoimmune POI, iron- and TGF-β-driven fibrosis in endometrioma, or adipokine- and insulin-driven remodeling in obesity. This subgroup heterogeneity has a direct clinical implication: a single elastographic cutoff, biomarker panel, or anti-fibrotic strategy is unlikely to apply equally across these subgroups, and future diagnostic and therapeutic development in this area will likely need to be stratified by disease subtype and, within PCOS and POI specifically, by phenotype, rather than treating ovarian fibrosis as one uniform target.

## 7. Clinical and Translational Implications

### 7.1. Ovarian Stiffness as a Potential Biomarker of Reproductive Aging

Endocrine and ultrasonographic measures, including anti-Müllerian hormone levels, follicle-stimulating hormone concentrations, antral follicle count, chronological age, and others, are currently the mainstays of ovarian reserve assessment in clinical reproductive medicine. Although these indicators are helpful for quantifying ovarian reserve, they do not always show qualitative deterioration of the ovarian microenvironment and do not capture the whole biological complexity of ovarian ageing [129]. Women with ostensibly normal endocrine profiles may possess compromised oocyte competency, suboptimal embryo development, or diminished implantation potential. This indicates that traditional evaluation methods may overlook significant elements of ovarian physiology. Recent research indicates that gradual structural and biomechanical remodeling of the ovarian stroma may occur prior to noticeable endocrine deterioration and may directly lead to reproductive failure [13].

Ageing and infertility cause gradual changes in the mechanical characteristics of ovarian tissue due to fibro-inflammatory remodeling. The heightened accumulation of collagen fibres, cross-linking of the extracellular matrix, activation of fibroblasts, persistent inflammatory infiltration, and vascular remodeling result in augmented stromal stiffness and disturbance of normal tissue elasticity [130]. The modifications may significantly impact vascular perfusion, granulosa cell functionality, follicular activation, oxygen transport, and granulosa–oocyte interaction. We infer that mechanical deterioration of the ovarian milieu may compromise reproductive capacity far earlier than the clinical manifestation of primordial follicle loss. By quantifying ovarian stiffness, we may be able to learn important things about tissue integrity, inflammation, and the architecture of the extracellular matrix [3]. In mice, atomic force microscopy has directly quantified this change: ovarian stiffness rises from 1.79 ± 0.08 kPa in reproductively young animals to 4.56 ± 2.03 kPa in reproductively old animals, roughly a 2.5-fold increase [22].

Recent advancements in elastographic imaging technology have prompted significant interest in ovarian biomechanics. Shear-wave elastography and other ultrasonic methods measure tissue elasticity by measuring the propagation velocity of mechanically produced shear waves in biological tissue. Fibrotic collagen-rich tissues exhibit more stiffness than typical elastic tissue, resulting in distinct elastographic features [131]. Preliminary research on ovarian stiffness suggests that women with polycystic ovary syndrome, diminished ovarian reserve, obesity-related infertility, and reproductive ageing may exhibit significant increases in ovarian rigidity compared to healthy reproductive control individuals; for example, shear-wave elastography in women with PCOS has shown mean ovarian stiffness of 14.9 ± 3.6 kPa versus 11.4 ± 2.3 kPa in controls, with a stiffness cut-off of 12.5 kPa distinguishing PCOS ovaries with reasonable diagnostic accuracy (AUC 0.816) [78].

The clinical uses of assessing ovarian stiffness extend well beyond basic diagnostic characterisation. Biomechanical evaluation of ovarian tissue has several potential applications, including estimating biological ovarian age, predicting ovarian response during controlled ovarian stimulation, identifying patients at high risk of adverse reproductive outcomes, and tracking disease progression in fibro-inflammatory reproductive disorders. Furthermore, the mechanical assessment of the ovarian cortex may provide indirect insights into extracellular matrix integrity, stromal fibrosis, inflammatory activity, and vascular remodeling, which are mostly unattainable by traditional ultrasonographic imaging. The correlation of biomechanical indicators with endocrine, metabolic, and molecular factors might significantly enhance personalized reproductive assessment [6].

The assessment of ovarian stiffness is inherently complicated due to the highly dynamic characteristics of ovarian physiology. The flexibility of tissue is affected by the menstrual cycle phase, follicular development, corpus luteum creation, vascular perfusion, inflammatory activity, extracellular matrix hydration, and local hormonal signaling [132]. Beyond this biological variability, technical factors further limit reproducibility. Shear-wave elastography measurements are known to vary meaningfully between operators depending on transducer angulation, probe pressure, and region-of-interest placement, a problem already documented for gynecological applications such as cervical elastography, where posterior and deeper structures show greater measurement variability than anterior, more superficial ones [132]. No consensus currently exists on the optimal timing of ovarian elastographic measurement within the menstrual cycle, and future studies will need to establish standardized operator training and a fixed cycle-day protocol before ovarian stiffness values can be compared reliably across patients or studies. The integration of elastographic imaging with molecular and cellular indicators may substantially enhance the translational significance of ovarian stiffness evaluation in the future. Possible fibro-inflammatory ovarian phenotypes can be identified by correlating biomechanical measurements with cytokine profiles in follicular fluid, extracellular matrix proteomics, markers of collagen turnover, oxidative stress, mitochondrial-function assays, and transcriptomic analysis [133]. Moreover, single-cell transcriptomics and spatial molecular mapping may elucidate the correlation between biomechanical changes and the cellular and molecular remodeling of the ovarian microenvironment. Multimodal techniques may potentially provide improved early diagnosis of stroma malfunction before the beginning of irreversible reproductive loss [134].

The biomechanical characterization of ovarian tissue may contribute to the comprehension of assisted reproductive outcomes. The augmentation of stroma stiffness may affect follicular development, mechanotransduction pathways, and vascular adaptation, hence destabilizing granulosa cell metabolism under regulated ovarian stimulation. Women with heightened ovarian stiffness may have atypical ovarian response, oocyte competency, or embryo developmental potential, while possessing an apparently sufficient endocrine reserve. Mechanical biomarkers may eventually aid in identifying individuals who need personalized stimulation methods or supplementary metabolic and anti-inflammatory therapies before IVF therapy. The acknowledgement of ovarian stiffness as a significant reproductive characteristic reflects a larger conceptual shift in reproductive medicine. Female infertility is now acknowledged as a condition that extends beyond mere endocrine failure, including gradual disturbances in tissue architecture, extracellular matrix integrity, vascular homeostasis, and biophysical signaling. Ovarian biomechanics may represent a significant feature of reproductive evaluation in the future, offering insights regarding ovarian ageing and infertility that have been hitherto overlooked [9].

### 7.2. Impact on Assisted Reproductive Technology and In Vitro Fertilisation Results

The structural integrity of the ovarian microenvironment seems to be increasingly significant in influencing the results of assisted reproductive technologies. Contemporary IVF procedures primarily emphasize optimal follicular recruitment and optimum oocyte yield with pharmaceutical gonadotropin stimulation. Clinical data indicates that the quantity of oocytes is not a reliable predictor of embryo viability, implantation success, or live birth rates. Women with ostensibly favourable ovarian reserve statistics may have suboptimal embryonic development or recurrent implantation failure, and conversely. Evidence is mounting that factors other than follicle count, such as fibro-inflammatory remodeling, extracellular matrix stiffness, oxidative stress, mitochondrial dysfunction, and stromal degradation, may significantly affect oocyte quality [135]. In a cohort of 325 infertile women undergoing IVF, those with low ovarian reserve, a clinical proxy for a degraded ovarian microenvironment, showed significantly lower total fertilization rates, oocyte yield, day-3 good-quality embryo rates, and blastocyst formation compared with women with normal or high ovarian reserve [135].

Numerous interrelated processes may link regulated stimulation to fibrotic alteration of the ovarian stroma and diminished ovarian response. The accumulation of extracellular matrix and collagen cross-linking modifies tissue flexibility and may physically restrict follicular growth during gonadotropin stimulation. Pathological stiffness of tissues hinders vascular adaptation and nutrient diffusion within the intra-follicular area, potentially affecting oxygen supply and metabolic equilibrium in developing follicles [25]. Altered biomechanical circumstances may further modify mechanotransduction pathways that regulate granulosa cell proliferation, mitochondrial function, cytoskeletal architecture, and follicular maturation. Consequently, follicles maturing in fibrotic ovarian conditions may have diminished developmental competence, while receiving sufficient hormonal stimulation [136].

Granulosa-cell dysfunction seems to be particularly significant for fibro-inflammatory ovarian transformation. Prolonged exposure to inflammatory cytokines, oxidative stress, hypoxia, metabolic instability, and aberrant extracellular matrix communication influences granulosa cells by altering steroidogenic function, mitochondrial metabolism, gap-junction communication, and cellular stress responses [137]. Compromised granulosa–oocyte metabolic collaboration may influence ATP availability, calcium equilibrium, spindle formation, and cytoplasmic maturation in the developing oocyte. Mitochondrial failure in granulosa cells may impair pyruvate metabolism and substrate transfer essential for meeting oocyte energy requirements. Such alterations may directly influence fertilization potential and embryonic developmental capability.

The mitochondrial integrity of the oocyte is notably vulnerable to persistent fibro-inflammatory remodeling. During meiotic maturation, fertilization, and early embryonic cleavage phases, oocytes rely significantly on effective oxidative phosphorylation. Ovarian fibrosis linked to persistent oxidative stress and inflammatory activation gradually impairs mitochondrial membrane potential, increases mitochondrial DNA damage, alters reactive oxygen species generation, and disrupts cellular redox equilibrium. Impaired mitochondrial quality-control mechanisms, such as defective mitophagy, may accelerate the accumulation of damaged mitochondria in ageing oocytes. These pathways may influence the embryo’s competence, despite the oocytes obtained during IVF cycles seeming morphologically normal [138].

The composition of follicular fluid increasingly seems to represent the biological condition of the ovarian milieu rather than serving just as a passive hormonal channel. Women with PCOS, endometriosis, obesity-related infertility, and reduced ovarian reserve exhibit elevated levels of inflammatory cytokines, extracellular matrix fragments, oxidative stress metabolites, iron-related products, advanced glycation end products, and extracellular vesicles containing inflammatory cargo. Variations in the molecular composition of follicular fluid may directly influence granulosa cell signaling, mitochondrial function, chromosomal integrity, and embryonic developmental potential. Consequently, the examination of follicular-fluid biomarkers will ultimately provide insights into fibro-inflammatory ovarian transformation and oocyte competency in assisted reproduction [139].

Among these biomarkers, advanced glycation end products (AGEs) warrant particular attention. AGEs are formed through the non-enzymatic glycation of proteins and lipids, accumulate with age and metabolic stress, and exert their pathogenic effects primarily through binding to their receptor, RAGE, on granulosa and stromal cells. AGE-RAGE engagement activates NF-κB and MAPK signaling, driving oxidative stress, granulosa cell dysfunction, and fibrotic remodeling, and has been implicated in premature ovarian failure, PCOS, and age-related ovarian decline. This positions follicular-fluid AGE levels not merely as a passive marker of metabolic exposure but as a proxy for an active fibro-inflammatory signaling pathway within the ovary, one that is also being explored as a therapeutic target through RAGE inhibitors and AGE-lowering interventions.

Vascular disease may also influence IVF results via ovarian fibrosis. Stromal stiffness and the gradual buildup of extracellular matrix may hinder angiogenesis and limit microvascular perfusion in stimulated ovaries. Gonadotropin stimulation, which prompts fast follicular development, may lead to diminished oxygen supply and regional hypoxia, thereby destabilising follicular metabolism and exacerbating oxidative damage [140]. Hypoxia-induced inflammatory activation may further hinder granulosa cell differentiation and disrupt the metabolic milieu essential for proper oocyte maturation. Furthermore, prolonged fibro-inflammatory ovarian dysfunction may indirectly influence endometrial receptivity. Oocytes subjected to inflammatory and oxidative ovarian microenvironments may yield embryos with modified metabolic activity, impaired developmental kinetics, or diminished implantation competence [118]. Moreover, systemic inflammatory and metabolic diseases, often linked to ovarian fibrosis, may influence immunological equilibrium in the endometrium, angiogenesis, decidualisation, and the signalling pathways pertinent to implantation. Consequently, reproductive failure in fibro-inflammatory ovarian illnesses may include a highly coordinated malfunction of the ovarian, embryonic, and endometrial compartments concurrently.

An additional consideration, particularly relevant to patients undergoing multiple treatment cycles, is whether controlled ovarian stimulation itself contributes to fibrotic progression. Repeated follicular rupture and wound repair are a recognized mechanical driver of stromal scarring, and mechanistic reviews specifically list repeated oocyte retrieval, alongside procedures such as cystectomy, among the causes of ovarian mechanical injury and subsequent fibrotic remodeling. In animal models, repeated cycles of ovarian stimulation produce a cycle-dependent increase in oxidative stress markers and structural ovarian damage. Whether this translates into clinically meaningful fibrotic progression in women undergoing several IVF cycles remains to be directly established, but it represents a plausible, cumulative mechanism worth monitoring in patients undergoing repeated stimulation.

Identifying ovarian stromal remodeling has significant implications for individualized reproduction strategy. Women exhibiting significant fibro-inflammatory ovarian dysfunction may exhibit varied responses to gonadotropin stimulation regimens and may gain advantages from supplementary antioxidant, anti-inflammatory, metabolic, or anti-fibrotic therapies prior to IVF therapy. The identification of ovarian fibrosis-associated characteristics may enhance patient classification and facilitate the creation of more tailored therapy strategies, optimizing both ovarian response and oocyte competence [141].

Recent years have seen a significant rise in interest about the optimization of the ovarian microenvironment before therapy. Investigated strategies for improving ovarian function and oocyte quality in specific patient populations encompass metformin, melatonin, mitochondrial-targeted antioxidants, platelet-rich plasma, extracellular vesicle-based therapies, anti-inflammatory agents, and extracellular matrix-modulating interventions [142]. Although most of the existing data is experimental and varied, these techniques indicate an increasing acknowledgement that maintaining stromal homeostasis may be a crucial factor in reproductive success during assisted reproduction. Future IVF techniques are likely to rely less on endocrine reserve evaluation and more on the incorporation of molecular, metabolic, inflammatory, and biomechanical characterisation of ovarian tissue. Comprehending the significance of fibro-inflammatory modification in follicular physiology, granulosa cell functionality, and oocyte competence might significantly enhance personalized reproductive care and facilitate the creation of physiologically focused fertility preservation techniques.

### 7.3. Novel Anti-Fibrotic and Microenvironment-Targeted Therapies

The increasing recognition of ovarian fibrosis as an active contributor to infertility has sparked interest in treatment approaches focused on maintaining or restoring stromal homeostasis. Contemporary assisted reproductive treatments primarily emphasize endocrine stimulation and ovulation induction, neglecting the structural deterioration of the ovarian microenvironment [6]. Recent experimental data indicates that the manipulation of inflammatory signaling, extracellular matrix modification, mitochondrial dysfunction, oxidative stress, and mechanotransduction pathways may partially alleviate ovarian fibrosis and enhance reproductive function. Ovarian stromal plasticity is becoming seen as a potentially adaptable biological process, rather than an irrevocable consequence of reproductive ageing [39].

The transmission of TGF-β is a very promising molecular target for anti-fibrotic intervention in ovarian tissue. Persistent activation of TGF-β/SMAD pathways promotes fibroblast proliferation, myofibroblast differentiation, collagen production, extracellular matrix crosslinking, and suppression of matrix breakdown processes [143]. Excessive activation of these pathways results in the progressive transformation of the ovarian stroma into a stiff fibro-inflammatory environment, characterized by reduced follicular support and vascular dysfunction. Experimental inhibition of TGF-β activation has shown decreased collagen formation and less tissue fibrosis across many organ systems. Comparable strategies may finally be pertinent to constraining stromal plasticity inside the ovary in age-related, inflammatory, or metabolically induced reproductive diseases [6].

Any translation of TGF-β inhibition to ovarian therapy, however, must contend with the fact that TGF-β family signaling is not simply pathological in this tissue. TGF-β and its relatives, including AMH and the oocyte-derived factors GDF9 and BMP15, are required for normal follicle activation, granulosa cell survival, cumulus expansion, and the periovulatory transition itself, and systemic TGF-β blockade in other clinical contexts has raised concerns about effects on fertility and pregnancy maintenance that have not been formally studied. This dual, concentration- and stage-dependent role means that a systemic anti-fibrotic strategy targeting TGF-β could plausibly impair ovulation and fertility in women of reproductive age even as it reduces stromal fibrosis, and any such approach would need ovary-sparing or highly localized delivery strategies rather than systemic inhibition.

The modulation of oxidative stress is a significant therapeutic approach in ovarian fibrosis. The persistent production of ROS exacerbates inflammatory activation, mitochondrial dysfunction, extracellular matrix remodeling, and cellular senescence within the ovarian microenvironment. Consequently, antioxidant agents that may stabilize mitochondrial function and restore redox equilibrium may disrupt the detrimental loop between oxidative damage and fibrosis advancement. Melatonin, coenzyme Q10, N-acetylcysteine, resveratrol, and mitochondria-targeted antioxidants have shown varying protective benefits in experimental reproductive models. Possible processes include less mitochondrial DNA damage, increased oxidative phosphorylation efficiency, inhibited inflammatory regulation, stable granulosa cell metabolism, and decreased fibroblast activation [144].

Metformin has been thoroughly investigated for its metabolic, anti-inflammatory, antioxidant, and anti-fibrotic properties. Besides boosting insulin sensitivity, metformin modulates AMP-activated protein kinase signaling, suppresses mTOR activation, reduces inflammatory cytokine production, promotes mitochondrial function, and affects pathways related to extracellular matrix remodeling [145]. Experimental findings indicate that metformin may mitigate age-related ovarian fibrosis and partly maintain stromal architecture in ageing animals. Metformin use in women with PCOS may activate the same pathways that boost reproductive results. Modulating metabolic stress pathways may be a crucial approach to mitigate fibro-inflammatory ovarian remodeling. The rising acknowledgement of ovarian fibrosis as a physiologically active and possibly alterable phenomenon has prompted heightened interest in innovative diagnostic and therapeutic strategies aimed at addressing stromal dysfunction, extracellular matrix remodeling, oxidative stress, and ovarian biomechanics [146]. It should be stressed, however, that no randomized controlled trials have evaluated metformin, melatonin, N-acetylcysteine, or any other agent discussed above with ovarian fibrosis or stromal biomechanics as a defined clinical endpoint; the supporting evidence for these compounds derives entirely from preclinical, animal, or mechanistic studies, or from human trials designed around unrelated endpoints such as metabolic parameters in PCOS. Table 3 summarizes emerging translational techniques and reflects this predominantly preclinical evidence base.

Beyond efficacy, the translational path for any of these strategies must explicitly address risk mitigation. Systemic modulation of TGF-β, oxidative-stress, or mTOR-related signaling carries a plausible risk of impairing normal follicle activation, granulosa-cell survival, or ovulation, since these same pathways support physiological reproductive function outside the fibrotic setting, as already noted for TGF-β above. Ovary-sparing or localized delivery, dose-ranging safety evaluation prior to any efficacy trial, and monitoring of reproductive endpoints, such as ovarian reserve markers, oocyte and embryo quality, and pregnancy outcomes, rather than fibrosis markers alone, would be necessary to exclude iatrogenic harm before any of these agents could be considered for women seeking to preserve or restore fertility. None of the compounds discussed in this section have been evaluated for reproductive safety in this specific context, and this absence of safety data should be regarded as a major barrier to clinical translation, independent of any efficacy signal.

Emerging diagnostic tools and therapeutic strategies aiming to evaluate or modulate ovarian fibrosis, extracellular matrix remodeling, inflammation, oxidative stress, and ovarian biomechanics in female infertility.

The localized delivery strategies invoked above remain, at present, largely conceptual for ovarian fibro-inflammatory disease and have not been validated in controlled human trials for this indication. Table 4 summarizes candidate approaches, drawn from the broader ovarian drug-delivery and nanomedicine literature, that could in principle be adapted to concentrate anti-fibrotic or regenerative agents within ovarian tissue while limiting systemic exposure.

These strategies are at a preclinical or early conceptual stage for the fibro-inflammatory indications discussed in this review and have not been tested for reproductive safety or efficacy against ovarian fibrosis in humans. Their inclusion here is intended to outline a future direction for translating the mechanistic targets described above (Table 3) into ovary-selective therapy, rather than to imply established clinical utility.

## 8. Discussion

Recent research indicates that ovarian fibrosis is an active biological process that directly influences reproductive ageing and female infertility, rather than only a passive histological outcome of ovarian senescence. The findings from our research collectively demonstrate that ageing ovaries undergo progressive extracellular matrix remodeling accompanied by chronic inflammation, stromal cell activation, modifications in tissue biomechanics, immune dysregulation, and vascular decline. These findings greatly expand upon the traditional understanding of ovarian ageing as a result of the gradual loss of follicles. The ovarian microenvironment seems to play a crucial role in maintaining the structural integrity essential for follicular dynamics, granulosa cell functionality, mitochondrial homeostasis, and oocyte competence.

Briley et al. presented one of the first robust pieces of evidence that reproductive ageing correlates with significant stromal fibrosis and collagen buildup in mammalian ovaries [55]. Their results indicated that fibrosis occurs gradually during reproductive ageing, rather than just at severe stages of ovarian failure. Umehara et al. subsequently reinforced the functional significance of this finding by showing that the buildup of fibrotic collagen inhibits follicular expansion and directly contributes to reproductive decline in mice [101]. The experimental findings revealed that targeted removal of fibrotic collagen may partly restore ovarian function and extend reproductive longevity in this murine model, therefore providing substantial evidence for a causal relationship between fibrosis and ovarian failure in mice; whether the same causal relationship holds in the human ovary has not been directly tested, since no equivalent collagen-depletion or anti-fibrotic intervention study has been conducted in women. Shen et al. elaborated on these results by highlighting that stromal remodelling alters the ovarian milieu via inflammatory activation, extracellular matrix buildup, vascular impairment, and disrupted intercellular communications [148]. Collectively, these investigations substantiate the conclusion that reproductive ageing correlates with the gradual alteration of the ovarian stroma into a physiologically detrimental milieu for follicular growth.

Amargant et al. and Parkes et al. substantially improved the understanding of the extracellular matrix composition in ovarian fibrosis [18,22]. Amargant et al. demonstrated that ovarian stiffness dramatically increases with age, primarily influenced by the organization of the collagen and hyaluronan matrix [22]. Meanwhile, Parkes et al. emphasized the significance of the transformation of hyaluronan-rich extracellular matrix in ageing ovarian tissue [18]. Dipali et al. demonstrated proteomic evidence of age-related alterations in extracellular matrix-enriched ovarian proteins [149]. Together, these data support the concept that ovarian fibrosis is not just a process of collagen buildup, but rather a dynamic reshaping of the ovarian matrisome. The interplay among these investigations is essential, as they jointly demonstrate that aging-related fibrosis is linked to extensive biochemical and biomechanical remodeling, which may modify tissue elasticity, follicular architecture, and cellular communication inside the ovarian cortex.

The studies of Shen et al., Isola et al., Tang et al., and Zhu et al. substantiate the notion that ovarian fibrosis develops within a chronic inflammatory milieu alongside reproductive ageing [5,148,150,151]. Lliberos et al. demonstrated a gradual inflammatory activation in ovarian ageing. Isola et al. discovered significant alterations in immune-cell populations and stromal-cell states in ageing ovaries by single-cell transcriptome analysis [152]. Tang et al. emphasized the significance of macrophage adaptability in ovarian reconfiguration and fibrosis, whereas Zhu et al. linked persistent low-grade inflammation to stromal dysfunction and extracellular matrix buildup [5,151]. Shen et al. integrated these data and proposed that chronic inflammatory activation directly contributes to fibroblast activation, vascular instability, and the gradual degradation of the stroma [9]. The findings indicate that ovarian fibrosis involves a fibro-inflammatory process influenced by chronic immunological dysregulation rather than only isolated extracellular matrix deposition.

Gu et al. and Edepli et al. elucidate the molecular foundation required to interpret the conversion of inflammatory and oxidative pathways into stromal fibrosis [6,7]. Both studies emphasize the critical importance of TGF-β signaling, fibroblast activation, extracellular matrix cross-linking, oxidative stress, and matrix breakdown dysfunction in the evolution of fibrosis. Gu et al. have comprehensively examined the function of TGF-β/SMAD signaling in the initiation of collagen production and the differentiation of myofibroblasts [7]. Edepli et al. associated these pathways with reproductive ageing, mitochondrial malfunction, and chronic inflammatory activation [6]. These investigations define oxidative stress not just as a consequence of ageing, but as an active enhancer of fibro-inflammatory transformation. Reactive oxygen species may enhance fibroblast activation, impair mitochondrial function, and perpetuate inflammatory communication, creating a detrimental loop that gradually undermines the homeostasis of ovarian tissue.

This perspective is also corroborated by the research of Isola et al., Dipali et al., and Zhu et al., suggesting that ovarian ageing affects not just follicles but other cellular compartments [5,149,150]. Isola et al. demonstrated synchronized transcriptome alterations in stromal, immunological, endothelial, and follicular cell populations inside ageing ovaries [153]. Dipali et al. discovered a comprehensive extracellular matrix and stress-related proteome remodeling, whereas Esencan et al. revealed fibro-inflammatory molecular markers in human follicular fluid during reproductive ageing [149,154]. Zhu et al. further underlined that chronic inflammatory remodeling affects both stromal integrity and follicular function. All things considered, our results lend credence to a systems-level model of ovarian ageing in which many processes, including inflammatory activation, immunological dysregulation, oxidative stress, and extracellular matrix remodeling, start simultaneously and work together to diminish reproductive capability.

Wang et al., Vasse et al., Amargant et al., and Umehara et al. jointly enhance the knowledge of ovarian physiology in relation to mechanobiology and mechanotransduction [20,89,101,141]. Wang et al. demonstrated that biomechanical communication pathways, including Hippo/YAP-TAZ signaling, integrin-mediated transcription, cytoskeletal remodeling, and tissue stiffness, significantly influence follicular activation and ovarian function [20]. Likewise, Vasse et al. emphasized the significance of ovarian biomechanics in the control of follicles [89]. Amargant et al. experimentally established that ovarian stiffness increases with age [22]. Umehara et al. subsequently showed that fibrotic stromal restriction functionally hindered follicular development [101]. These findings together indicate that follicles react not only to endocrine stimulation but also to the physical attributes of the adjacent ovarian tissue. Consequently, excessive rigidity of the extracellular matrix may negatively affect folliculogenesis via modified mechanotransduction signaling, aberrant cytoskeletal tension, and restricted follicular growth.

The ovarian remodeling in PCOS is well exemplified by the works of Hopkins et al., He et al., Wang et al., and Gu et al. [7,20,78,155]. Nevertheless, only a limited number of research has investigated the function of ovarian tissue biomechanics in PCOS. Hopkins et al. discovered that elevated androgens lead to extracellular matrix dysregulation and the activation of pro-fibrotic signaling pathways in the ovary, whereas He et al. evidenced heightened ovarian stiffness in women with PCOS by shear-wave elastography [78,155]. Wang et al. added to the mechanotransduction perspective by linking tissue stiffness to alterations in Hippo/YAP activation and follicular dynamics, while Gu et al. elucidated the molecular pathways connecting inflammation, fibrosis, and extracellular matrix remodeling [7,20]. The findings of these investigations indicate that PCOS should not be regarded just as an endocrine–metabolic disorder. Hyperandrogenism, inflammation, extracellular matrix remodeling, and modified ovarian biomechanics seem to be closely interconnected in polycystic ovaries. Consequently, stromal stiffness may directly influence follicular arrest and diminish oocyte competence in PCOS. It should be noted that these mechanobiology findings have not been stratified by Rotterdam phenotype; as discussed in Section 6.1, the hyperandrogenic phenotypes (A and B) are expected to carry the greatest fibro-inflammatory burden, and whether these findings extend to the normoandrogenic phenotype D remains to be established.

Briley et al. illustrated a gradual cortical fibrosis associated with ageing, whereas Amargant et al. revealed that ovaries exhibit increased mechanical stiffness with age [22,55]. Wang et al. emphasized the significance of Hippo/YAP mechanotransduction pathways in follicular activation, whereas Umehara et al. showed that the decrease in fibrosis enhances ovarian function. In summary, our data indicate that excessive buildup of extracellular matrix may disrupt primordial follicle activation via biomechanical dysregulation, affecting cytoskeletal tension, YAP/TAZ localization, and ovarian cortex architecture [20,101]. Consequently, reproductive ageing may entail a gradual destabilization of the biomechanical conditions essential for physiological follicular homeostasis. McCloskey et al. proved that metformin reduces ovarian fibrosis in ageing models, whereas Landry et al. showed that metformin influences fibroblast, myofibroblast, and immune cell populations in ageing ovarian tissue [147,156]. Umehara et al. showed direct proof that collagen-targeting techniques may enhance ovarian function [101]. Amargant et al. established the advantageous effects of anti-fibrotic therapies on ovarian ageing [22]. The results of these studies together challenge the notion that stromal fibrosis represents an irreversible culmination of reproductive loss. Ovarian remodeling seems to be physiologically dynamic and possibly responsive to metabolic, anti-inflammatory, and anti-fibrotic treatment strategies.

The researchers Εsencan et al., Dipali et al., Gu et al., and Edepli et al. provided evidence of a robust correlation between mitochondrial malfunction, oxidative stress, and ovarian fibrosis [6,7,149,154]. Εsencanet al. revealed inflammatory and oxidative modifications in human follicular-fluid communication, whereas Dipali et al. identified proteome reconfiguration including alterations in stress-response proteins and extracellular matrix pathways [6,149]. Gu et al. demonstrated that oxidative stress induces fibroblast activation and extracellular matrix deposition, whereas Edepli et al. associated chronic inflammatory transformation during reproductive ageing with mitochondrial failure [7,149]. The findings of these investigations clearly indicate that reactive oxygen species serve not only as indicators of ovarian ageing but also as active mediators of fibrosis advancement and stromal dysfunction. Mitochondrial instability, oxidative stress, extracellular matrix remodeling, and chronic inflammation seem to be intricately linked with reproductive decline.

He et al. established the viability of quantifying ovarian stiffness in patients with PCOS [157]. Wang et al. and Vasse et al. elucidated the biological mechanisms by which biomechanical characteristics influence follicular physiology and ovarian function [20,89]. Because existing ovarian reserve measures mostly represent follicular abundance rather than stroma quality and tissue integrity, these results have important therapeutic implications. Thus, biomechanical examination may finally provide insights into fibrosis load, extracellular matrix architecture, inflammatory remodeling, and mechanotransduction status within ovarian tissue, thereby complementing endocrine evaluation.

Mechanotransduction pathways may potentially serve as direct therapeutic targets in reproductive medicine. Modulation of Hippo/YAP-TAZ signaling has been shown to affect follicular activation by mechanical manipulation of ovarian tissue. As a result, forthcoming treatment options aimed at restoring physiological ovarian biomechanics or diminishing pathological extracellular matrix stiffness may provide innovative methods that improve follicular dynamics and safeguard ovarian reserve. The pharmacological manipulation of mechanosensitive signaling pathways, extracellular matrix cross-linking enzymes, or cytoskeletal structure is currently extremely experimental; however, it is a swiftly advancing domain with considerable translational promise.

Cellular senescence is a promising therapeutic target in ovarian fibrosis and reproductive ageing. Senescent stromal fibroblasts and granulosa cells are essential facilitators of chronic inflammatory activation and extracellular matrix buildup via the release of pro-fibrotic mediators, cytokines, matrix-remodeling enzymes, and reactive oxygen species. Senolytic treatments, intended to selectively eradicate senescent cells, have shown promising outcomes in experimental ageing models and may be pertinent to reproductive ageing and fibrosis-related infertility in the future. Modulating senescence-associated pathways to restore tissue regeneration ability may enhance ovarian microenvironmental integrity and prolong reproductive life.

Therapies using extracellular vesicles have garnered significant scientific attention in recent years. Extracellular vesicles generated from mesenchymal stem cells are abundant in microRNAs, proteins, lipids, anti-inflammatory agents, and regenerative signaling molecules that may influence fibrosis, oxidative stress, angiogenesis, mitochondrial equilibrium, and tissue repair. Experimental research in reproduction indicates that extracellular vesicles may boost ovarian function by controlling inflammatory signaling, restoring mitochondrial activity, boosting angiogenesis, and diminishing extracellular matrix remodeling. Although mostly experimental, these methods underscore the rapidly evolving treatment landscape aimed at restoring ovarian microenvironmental homeostasis.

Vascular-targeted treatments may potentially be pertinent to ovarian fibrosis. The gradual buildup of extracellular matrix and increased stromal stiffness might hinder angiogenesis and tissue perfusion, resulting in localized hypoxia and metabolic instability in ovarian tissue. Therefore, by regulating angiogenesis, safeguarding the endothelium, or addressing hypoxia, microvascular integrity may be restored, potentially enhancing follicular metabolism and the fibro-inflammatory process. As a result, enhanced comprehension of ovarian vascular remodeling may provide more treatment alternatives for fertility maintenance.

Four methodological issues warrant explicit comment before this evidence is applied clinically: the grading of evidence strength, the proportion of findings derived from human rather than animal or in vitro data, the standardization of criteria used to define and quantify ovarian fibrosis, and the risk-mitigation requirements of any future therapeutic application. The first three are addressed in this and the following paragraph, and the fourth in Section 7.3. Taken together, however, this body of evidence has important limits that temper how it should be interpreted. The great majority of mechanistic and causal data, including the collagen-removal and anti-fibrotic intervention studies discussed above, come from murine models; human evidence remains largely correlational, drawn from cross-sectional histology, elastography, or proteomics rather than from longitudinal or interventional studies capable of establishing causation. The methods used to define and quantify ‘fibrosis’ also vary considerably across the cited studies, ranging from collagen histology to shear-wave stiffness to proteomic signatures, and these different metrics have not been systematically cross-validated against one another, which complicates direct comparison of findings across the literature. A further open question is directionality: it remains unclear in humans whether stromal fibrosis is a primary driver of follicular loss and reproductive decline, or whether it develops secondarily as follicles are lost and the ovarian cortex is progressively replaced by stroma, a distinction the current evidence base cannot yet resolve. Any translational optimism regarding anti-fibrotic or mechanotransduction-targeted therapies should therefore be weighed against the largely preclinical, associative nature of the supporting data.

Weighing the evidence by category clarifies how much confidence each claim in this review actually warrants. That ageing ovaries accumulate collagen and stiffen is well established, having been shown repeatedly in both animal models and direct human tissue measurements. That this stiffening is accompanied by chronic low-grade inflammation and altered immune-cell composition is similarly well supported across species. By contrast, the claim that fibrosis is a primary causal driver of follicular loss, rather than a parallel or downstream process, remains a hypothesis rather than an established fact, given the near-total reliance on animal intervention studies for causal evidence. The molecular convergence proposed across PCOS, endometriosis, obesity, and premature ovarian insufficiency onto shared TGF-β, Hippo/YAP, and inflammatory pathways is a plausible and increasingly well-supported unifying model, but it has been assembled from disease-specific studies that were not designed for direct cross-condition comparison. Finally, the anti-fibrotic, mechanotransduction-targeted, and biomarker-based clinical applications discussed in Section 7 and Section 8 remain firmly in the preclinical or early proof-of-concept stage, and none should be read as having established clinical utility.

An alternative interpretation also deserves consideration. Across other organ systems, fibrosis is well established as an initially adaptive, protective response, one that contains tissue damage and limits the spread of acute inflammation, and only becomes pathological when the injury is prolonged or repeated. It is plausible that some degree of ovarian stromal fibrosis, particularly in the context of repeated ovulatory injury or localized inflammatory insults such as endometriotic lesions, similarly functions to wall off and limit chronic inflammation rather than acting purely as a driver of follicular loss. Under this view, fibrosis and reproductive decline would be better understood as parallel consequences of an underlying inflammatory or metabolic insult rather than fibrosis itself being the principal pathogenic step, a distinction with direct implications for whether anti-fibrotic therapy would help or potentially remove a protective barrier. Distinguishing a protective, containment-oriented fibrotic response from a maladaptive, function-limiting one in the ovary has not yet been directly addressed and represents an important direction for future research.

This containment-versus-maladaptive distinction points toward a staged trajectory rather than a fixed state. A reactive, early stage would be dominated by localized, containment-oriented fibrosis; an intermediate stage would see fibrosis begin to spread and measurably raise ovarian stiffness ahead of any hormonal change; and a late stage would see fibrosis become self-sustaining and directly drive follicular loss. Such a staged model is speculative and has not been tested directly, but it offers a way to organize the cross-sectional findings discussed throughout this review into a coherent, testable trajectory. We return to it in Section 9.

Future anti-fibrotic therapies will likely need a multifaceted strategy, integrating metabolic, inflammatory, oxidative, biomechanical, and molecular interventions rather than relying just on endocrine regulation. Ovarian fibrosis seems to be an increasingly complex pathological process characterized by persistent interactions among inflammation, mitochondrial failure, extracellular matrix remodeling, cellular senescence, vascular impairment, and mechanotransduction anomalies. Consequently, effective treatment intervention may need the concurrent restoration of several elements of ovarian microenvironmental homeostasis instead of focusing on a single isolated mechanism. The recognition of ovarian fibrosis as a potentially reversible and physiologically dynamic phenomenon has initiated a significant new avenue in reproductive medicine. The primary objectives of fertility preservation and individualized reproductive medicine are maintaining stromal integrity, ensuring the flexibility of the extracellular matrix, stabilizing mitochondria, and achieving biomechanical equilibrium.

Evidence from clinical populations outside the fibrosis literature itself lends indirect support to this biomarker-based direction. A recent study of an egg donor cohort identified an AMH threshold associated with donor eligibility and reproductive outcome, reinforcing that follicular reserve, rather than age alone, drives donation success [158]. Comparable AMH and antral-follicle-count thresholds have been proposed to guide patient selection for oocyte cryopreservation after in vitro maturation [159] and have been linked to clinical pregnancy rates after IVF-embryo transfer [160]. If endocrine markers such as AMH already guide major reproductive decisions in these settings, non-invasive structural markers of the ovarian microenvironment, such as ovarian stiffness and extracellular matrix integrity, could add complementary, mechanistic information beyond follicle counting alone, consistent with the translational rationale of this review.

Some recent studies push this question further: can ovarian fibrosis actually be reversed, not just slowed? In mice, enzymatically removing collagen from the ovary worked fast. Follicles resumed developing and ovulation returned within days. But natural pregnancies and litter size did not improve. Cutting stiffness alone was not enough to restore fertility [101]. A separate approach, low-dose systemic anti-fibrotic treatment, slowed age-related fibrosis and kept more of the stromal architecture intact in older mice [141]. Human data is still very limited. One small trial gave the antifibrotic drug finerenone to 14 women with premature ovarian insufficiency. All 14 developed follicles, and seven produced mature eggs, with quality comparable to age-matched women without the condition, after three to seven months of oral treatment [161]. A separate phase 1 trial transplanted amniotic epithelial cells into the ovarian artery in women with premature ovarian failure. It was reported as safe, and some participants regained hormone levels and menstrual function, though this approach targets follicles and hormones rather than fibrosis directly [162]. Both trials are small, uncontrolled, or single-arm. Before any of this reaches the clinic, it will need controlled trials with real reproductive outcomes, not just follicle counts, and longer follow-up.

**Figure 2:**
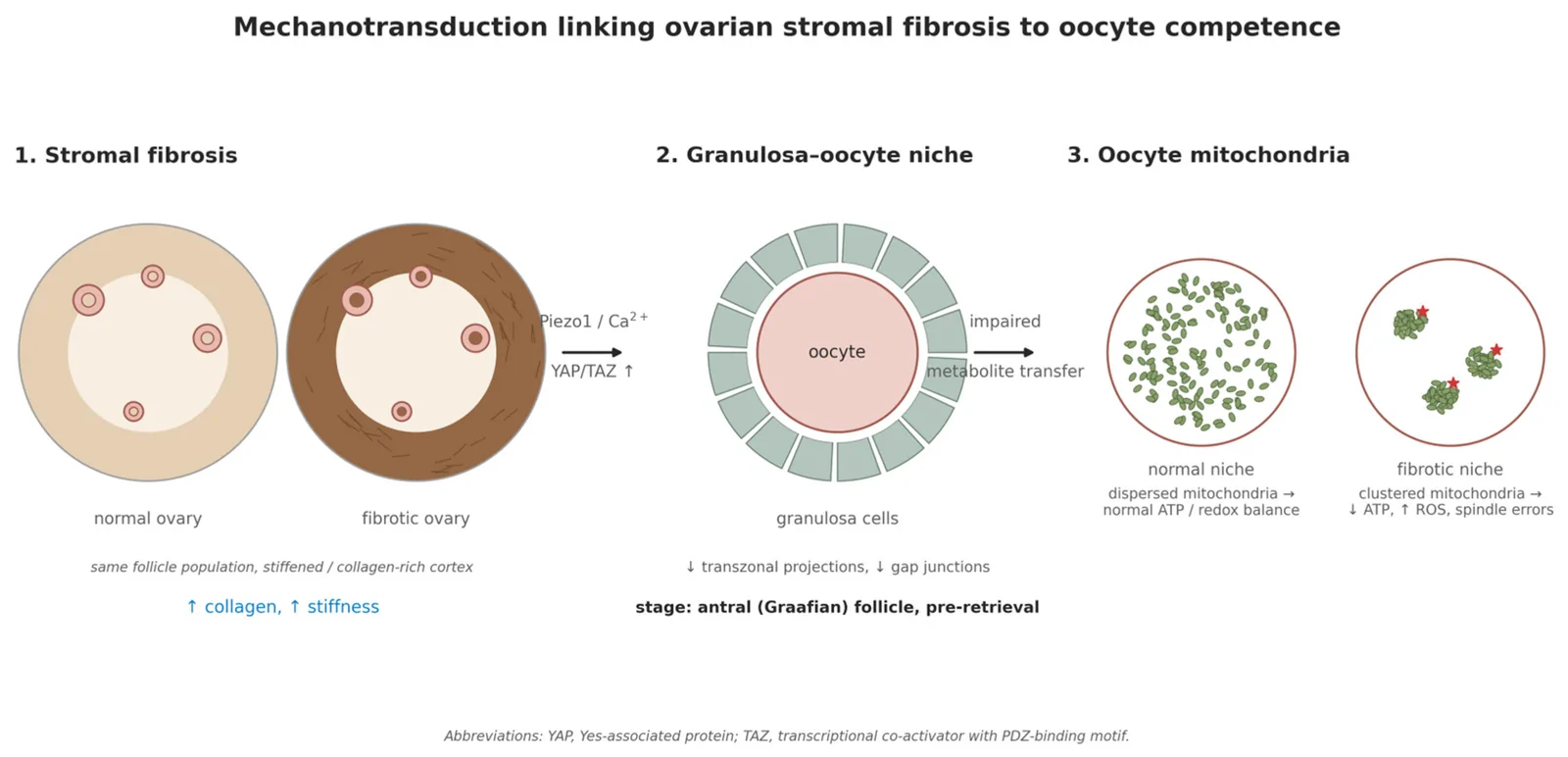
Mechanotransduction linking ovarian stromal fibrosis to oocyte competence. (1) In the normal ovarian cortex, follicles develop within a loosely organized collagen matrix; with ageing, PCOS, diminished ovarian reserve, endometriosis, or obesity, collagen deposition and cross-linking increase, producing a stiffer, fibrotic cortex [3,9,63]. (2) At the granulosa–oocyte interface, this mechanical stiffening is sensed through the Piezo1 channel and calcium influx, promoting nuclear translocation of YAP/TAZ and reducing transzonal-projection and connexin-43 gap-junctional connectivity between granulosa cells and the oocyte [26,64,67,68,82]. (3) The resulting disruption in metabolite and calcium transfer compromises oocyte mitochondrial membrane potential and redox balance, favours reactive-oxygen-species accumulation and meiotic-spindle errors, and is associated with reduced oocyte developmental competence [82,83,85]. Abbreviations: YAP, Yes-associated protein; TAZ, transcriptional co-activator with PDZ-binding motif.

Mechanotransduction and inflammatory signaling do not act as separate systems in the fibrotic ovary. They reinforce each other through direct molecular links. Matrix stiffness activates YAP and TAZ, as described above. This activation increases fibroblast sensitivity to TGF-β and strengthens the pro-fibrotic transcriptional programme it drives [163]. Integrins add a second, mechanical layer to this loop. Tension transmitted through integrin–ECM contacts physically releases active TGF-β from its latent complex, linking mechanical force directly to cytokine activation. This happens independently of upstream inflammatory triggers [164]. In the ovary, this means the stiffening events described in Section 5 and the cytokine-driven remodeling described in Section 3, Section 4 and Section 6 are not parallel processes. They form a single feed-forward circuit: collagen accumulation raises stiffness, stiffness activates YAP/TAZ and integrin-mediated TGF-β release, and this reinforces fibroblast activation and further collagen deposition. Table 1 and Figure 2 summarize the components of this network. Most of the direct evidence for this specific crosstalk comes from other fibrotic organs, particularly lung, liver, and kidney. Ovary-specific confirmation of this mechanical-inflammatory loop is still limited and remains an open question for future work.

Recent single-cell profiling adds detail to this picture. In the mouse ovary, the immune compartment is innate-dominated early in reproductive life, with macrophages, neutrophils, and NK-like innate lymphoid cells making up most of the resident population. With age, a distinct T-cell population, double-negative T cells, expands substantially, from roughly five percent to over a third of the total immune compartment. This represents a compositional shift toward adaptive immunity, layered on top of the macrophage-centred remodeling already described above [165]. Somewhat unexpectedly, aged immune cells in this dataset showed reduced per-cell cytokine and chemokine output, alongside increased expression of receptors that recognize senescent cells, interpreted by the authors as a shift toward damage clearance rather than classic pro-inflammatory activity [165]. This does not fully align with the tissue-level rise in inflammatory cytokines described earlier in this review, and the two findings should be read as complementary rather than reconciled: total inflammatory burden in the ovary can still rise with age even if the average activity of each immune cell falls, simply because more immune cells are present overall. A related picture comes from Isola et al., who reported that resident macrophages decline while monocyte-derived, alternatively activated macrophages associated with fibrosis increase, alongside accumulation of type 1 and type 17 T lymphocytes [150]. Together, these findings support a staged model. Innate immune remodeling, largely macrophage-driven, dominates the earlier phase of ovarian ageing. A shift toward adaptive, T-cell-mediated immunity becomes more prominent later. This staged model comes entirely from mouse data. Whether the same sequence occurs in the human ovary is not yet known, and confirming it is a clear priority for future work.

The evidence summarized in this review draws on tissue and measurements collected in very different ways, and this heterogeneity is itself a limitation worth stating plainly. Human ovarian tissue in the cited studies comes from cortical biopsies taken for fertility preservation, from oophorectomy specimens removed for unrelated indications, and, less often, from postmortem samples; these sources differ in patient age, underlying diagnosis, and how the tissue was fixed and processed, any of which can affect collagen and extracellular matrix measurements. Where in the ovary the sample was taken matters too. Cortical and medullary regions differ in baseline collagen density, and not every cited study reports precisely which region was sampled. Animal studies add further variability: mouse strain, the age cutoffs used to define young and old, and whether stiffness was measured on excised tissue or in situ can all shift the absolute values reported. The stiffness figures cited earlier in this review illustrate the point directly. The mouse data come from atomic force microscopy on excised ovarian tissue, an ex vivo mechanical test [22], while the human PCOS data come from transvaginal shear-wave elastography performed in vivo [78]. These are not directly comparable measurements of the same physical property, and this review has treated them as separate lines of evidence for that reason, not as interchangeable values. None of this changes the overall direction of the findings, but it does mean that absolute stiffness values should not be compared across studies without accounting for how and where the tissue or measurement was obtained.

## 9. Conclusions

Ovarian fibrosis is not a passive result of ageing. The evidence here shows it as an active process in reproductive ageing and female infertility. Several changes drive this: extracellular matrix remodeling, collagen buildup, chronic inflammation, oxidative stress, mitochondrial dysfunction, vascular compromise, and altered tissue biomechanics. Together they reshape the ovarian microenvironment. They affect follicle activation, granulosa-cell behaviour, and oocyte mitochondrial function through Hippo/YAP-linked mechanotransduction. The same pathways recur across different conditions: polycystic ovary syndrome, obesity-related infertility, reduced ovarian reserve, premature ovarian insufficiency, and endometriosis. TGF-β signaling, macrophage activation, extracellular matrix buildup, and mechanotransduction abnormalities appear in all of them, despite different starting causes. This supports the fibro-inflammatory ovary as a unifying framework. It links endocrine, metabolic, inflammatory, and biomechanical aspects of female infertility.

As introduced in Section 8, we propose a staged model of the fibro-inflammatory ovary, building on the distinction there between containment-oriented and maladaptive fibrosis. In the early stage, fibrosis is reactive. It stays localized and limits inflammatory injury. Follicular reserve is not yet affected. In the intermediate stage, fibrosis spreads. It impairs mechanotransduction and vascular perfusion. Ovarian stiffness rises on elastography, even when hormone levels look normal. In the late stage, fibrosis becomes self-sustaining. It drives further inflammation and follicular loss on its own, independent of the original trigger. Anti-inflammatory and metabolic treatments should work best in the early stage. Anti-fibrotic or mechanotransduction-targeted therapy becomes appropriate only later, once fibrosis reaches the intermediate or late stage. This model needs testing. A prospective human cohort, combining elastography, inflammatory markers, and follicular-reserve data, could test it directly. That would let the field move from describing fibro-inflammatory change to timing treatment against it.

These findings matter for the clinic. Current assessment of ovarian reserve relies mainly on follicle counts, from hormone levels and ultrasound. The structural and biomechanical side of ovarian quality is largely ignored. Adding ovarian stiffness, extracellular matrix biomarkers, and inflammatory profiles could sharpen this picture. It may allow a more precise, personalized fertility evaluation. Preclinical studies also suggest fibrosis can be treated. Anti-fibrotic agents, metabolic drugs, oxidative-stress regulators, extracellular matrix-targeted approaches, and mechanotransduction-directed strategies all show promise. Restoring balance in the ovarian microenvironment might improve follicular activity and reproductive outcomes, beyond hormone stimulation alone. Human translation, though, remains limited by the barriers discussed above.

Future work should focus on three priorities. First, directionality. Does fibrosis cause follicular loss, or follow it? Only longitudinal human studies can answer this, linking stromal remodeling to IVF outcomes, oocyte competence, and reproductive ageing over time. Cross-sectional or animal-only studies cannot settle the question. Second, near-term steps that are already feasible: standardizing elastography protocols, validating follicular-fluid and serum biomarkers against clinical outcomes, and applying single-cell and spatial transcriptomics to human ovarian tissue already collected through fertility-preservation programs. Third, and hardest, therapeutic translation. Anti-fibrotic and mechanotransduction-targeted therapies still lack human trial data. Safety concerns remain, such as the TGF-β/ovulation conflict discussed earlier. Local, ovary-specific delivery is still difficult. Early-phase human safety studies are needed before efficacy can even be tested. These three priorities should be addressed in order, not all at once. That is the clearest path from describing ovarian fibrosis to treating it.

Progress in this area will depend on collaboration across fields that do not always work together. Reproductive biologists, biomechanical engineers, immunologists, and clinical embryologists each hold part of the picture, from ovarian tissue mechanics to inflammatory signaling to fertility outcomes. Elastography and other biomechanical tools need engineering input to standardize. Single-cell and spatial transcriptomic data need computational expertise to interpret at scale. And any anti-fibrotic strategy will need reproductive endocrinologists and fertility specialists to test it safely in patients who still want to conceive. Bringing these groups together, rather than each working in isolation, is likely the fastest route from the mechanisms described in this review to something a patient can actually be offered in the clinic.

## Figures and Tables

**Table 1:** Major Molecular and Cellular Drivers of Ovarian Fibrosis in Female Infertility.

Pathway/Mechanism	Main Molecular Mediators	Biological Effect on Ovary	Associated Infertility Conditions
TGF-β/SMAD signaling	TGF-β, SMAD2/3	Fibroblast activation, collagen deposition	Aging, DOR, POI, Endometriosis [6,7]
Hippo/YAP-TAZ dysregulation	YAP, TAZ	Abnormal follicular activation	Aging, PCOS [67,68]
Oxidative stress	ROS, mitochondrial dysfunction	ECM remodeling, DNA damage	Aging, Obesity [54,56]
Chronic inflammation	TNF-α, IL-6, IL-1β	Fibro-inflammatory remodeling	PCOS, Endometriosis [87,88]
Macrophage activation	M1 macrophages	Cytokine amplification, fibrosis	Aging, PCOS [47,89]
ECM accumulation	Collagen I/III, fibronectin	Increased stiffness	All conditions [23,25]
Vascular dysfunction	HIF-1α	Hypoxia, metabolic stress	DOR, Endometriosis [90,91]
Mechanotransduction abnormalities	Integrins, focal adhesions	Altered granulosa-cell signaling	Aging, PCOS [64,92]

Abbreviations: TGF-β, transforming growth factor beta; SMAD, mothers against decapentaplegic homolog (SMAD family signal transducer); YAP, Yes-associated protein; TAZ, transcriptional co-activator with PDZ-binding motif; ROS, reactive oxygen species; ECM, extracellular matrix; TNF-α, tumor necrosis factor alpha; IL-6, interleukin-6; IL-1β, interleukin-1 beta; M1, classically activated (pro-inflammatory) macrophage phenotype; HIF-1α, hypoxia-inducible factor 1-alpha; DOR, diminished ovarian reserve; POI, premature ovarian insufficiency.

**Table 2:** Fibro-Inflammatory and Biomechanical Alterations Across Female Infertility Disorders.

Disorder	Fibrosis	ECM Remodeling	Oxidative Stress	Inflammation	Ovarian Stiffness	Main Reproductive Consequence
Reproductive aging	Severe	High collagen accumulation	Increased	Chronic inflammaging	Increased	Decline in oocyte competence [55,101]
PCOS	Stromal hyperplasia/fibrosis	Excess collagen I/III	Increased	Chronic low-grade	Increased	Follicular arrest [92,94]
DOR/POI	Cortical fibrosis	ECM disorganization	Severe	Chronic	Increased	Reduced ovarian response [90,102]
Endometriosis	Pericortical fibrosis	TGF-β-driven remodeling	Severe	High inflammatory activity	Increased	Poor oocyte quality [103,104]
Obesity	Diffuse stromal remodeling	Adipokine-mediated	Lipotoxic oxidative stress	Metabolic inflammation	Probable increase	Reduced embryo competence [105]

Abbreviations: ECM, extracellular matrix; PCOS, polycystic ovary syndrome; DOR, diminished ovarian reserve; POI, premature ovarian insufficiency.

**Table 3:** Potential Diagnostic and Therapeutic Strategies Targeting Ovarian Fibrosis and Stromal Dysfunction.

Strategy	Target	Proposed Biological Effect	Current Evidence
Ovarian elastography	Tissue stiffness	Detection of biomechanical alterations	Early clinical evidence [78,131]
Anti-fibrotic therapy	Collagen deposition	Reduced stromal fibrosis	Experimental [6,101]
Metformin	Fibro-inflammatory signaling	Reduced fibrosis/inflammation	Preclinical evidence [145,147]
Antioxidants	ROS/mitochondrial dysfunction	Reduced oxidative injury	Experimental [144]
Hippo pathway modulation	YAP/TAZ signaling	Controlled follicular activation	Experimental [67,68]
ECM-targeted therapies	Matrix remodeling	Restoration of stromal elasticity	Preclinical [143]
Anti-inflammatory interventions	Cytokine signaling	Reduced chronic remodeling	Experimental [6,146]

Abbreviations: ECM, extracellular matrix; ROS, reactive oxygen species; YAP, Yes-associated protein; TAZ, transcriptional co-activator with PDZ-binding motif.

**Table 4:** Candidate Ovary-Targeted Delivery Strategies for Future Anti-Fibrotic and Regenerative Interventions.

Strategy	Target/Mechanism	Proposed Advantage
Direct intraovarian/stromal injection	Deposition of the agent directly into ovarian stroma	Local tissue concentration with reduced systemic exposure
Nanoparticle-based ovarian tropism	Passive nanoparticle accumulation in ovarian and follicular tissue during ovulation	Potential for ovary-selective drug concentration at reduced systemic dose
Nanomaterial-based carriers for ovarian disease	Encapsulation and sustained local release of anti-inflammatory/anti-fibrotic agents	Improved local bioavailability, reduced dosing frequency
Next-generation ovary-targeting technologies (e.g., ligand-functionalized nanocarriers)	Tissue-specific targeting of ovarian follicular structures	Improved reproductive safety profile versus systemic administration

## Data Availability

No new data were created or analyzed in this study. Data sharing is not applicable to this article.
